# Supplementary material for: Topography-aware optimal transport for alignment of spatial omics data
Source: Cell Rep Methods. 2026 Mar 30;6(4):101373. doi: 10.1016/j.crmeth.2026.101373 (PMC13107060; doi:10.1016/j.crmeth.2026.101373)
Supplement: Document S1. Figures S1–S6 [file mmc1.pdf]

Cell Reports Methods, Volume 6

Supplemental information

Topography-aware optimal transport for alignment of spatial omics data

Francesco Ceccarelli, Pietro Liò, Julio Saez-Rodriguez, Sean B. Holden, and Jovan Tanevski

SUPPLEMENTAL INFORMATION
Topography Aware Optimal Transport for Alignment of Spatial Omics Data

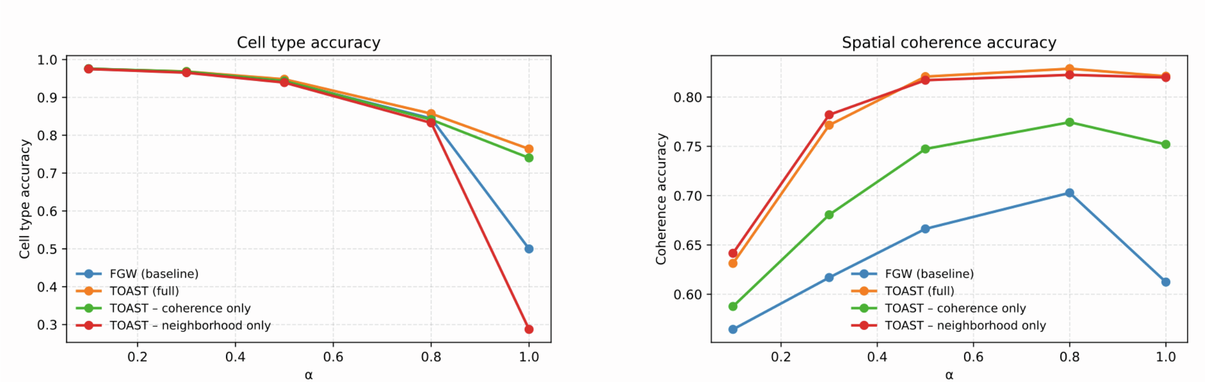

Figure S1: Spatial terms ablation study, related to **Figure 2**. Comparison between standard FGW, TOAST with both spatial coherence and neighborhood consistency, TOAST with only spatial coherence and TOAST with only neighborhood consistency.

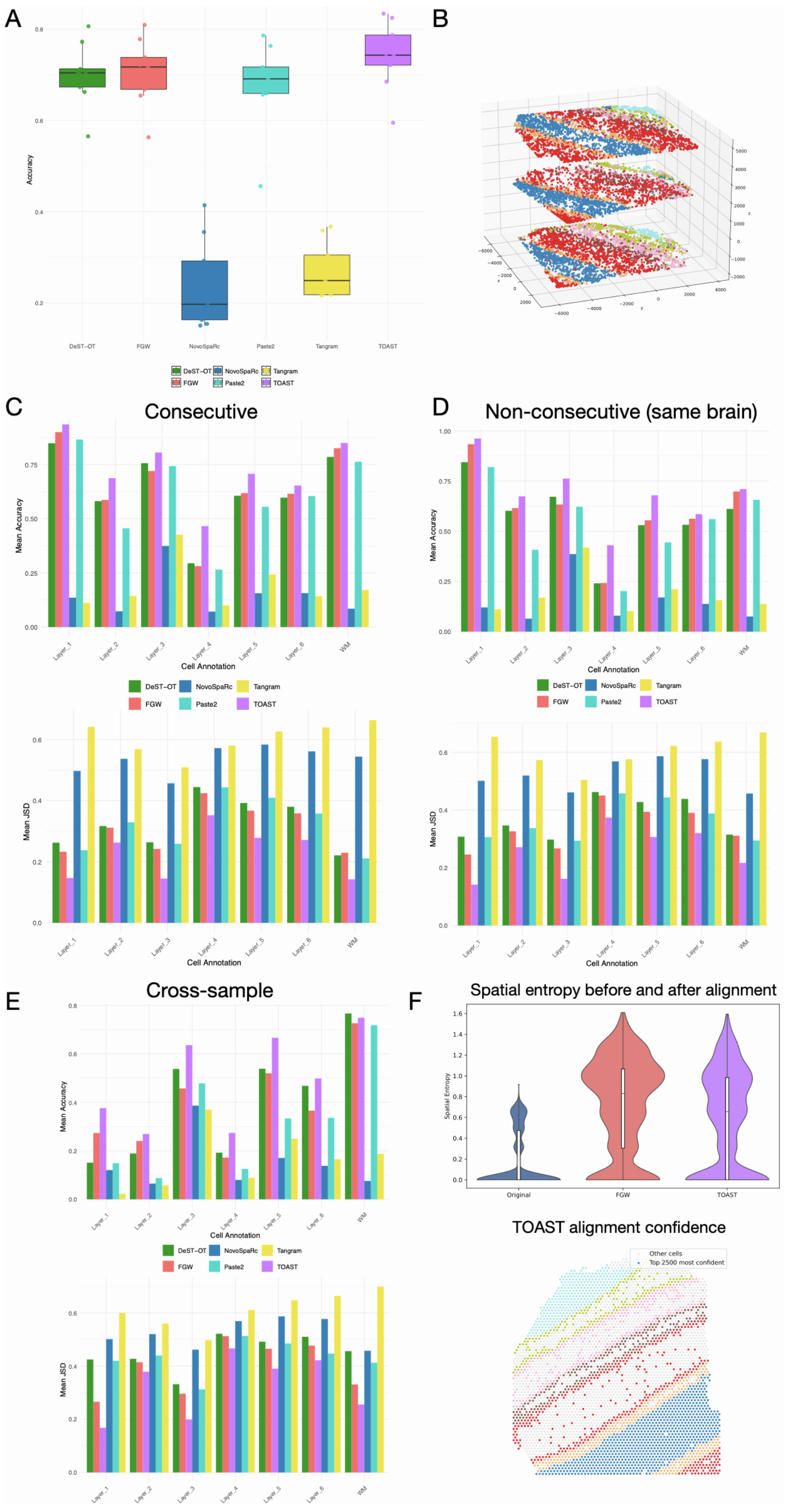

Figure S2: TOAST alignment on DLPFC data, related to **Figure 3**. **A.)** Quantitative comparison of pairwise alignment across consecutive slices for FGW, DeST-OT, Tangram, Paste2, NovoSparc and TOAST with cell labels obtained from unsupervised clustering. **B.)** Stacked 3D alignment of tissue slices of a DLPFC sample after alignment with TOAST. **C.)** Quantitative comparison of pairwise alignment across all consecutive, **D.)** non-consecutive, and **E.)** cross-sample slices in terms of accuracy and Jensen-Shannon divergence. **F.) Upper panel**: Violin plots showing the distribution of spatial entropy for the source slice prior to alignment and after reconstruction using FGW and TOAST. TOAST preserves the original spatial organization more faithfully than FGW. **Lower panel**: TOAST alignment confidence for a pairwise alignment. We display the top 2,500 most confidently aligned cells (colored by ground-truth annotation), while lower-confidence cells are shown in light gray. Distinct cell populations exhibit different confidence profiles. These maps guide downstream analyses by indicating which aligned cells are reliable, and which warrant further biological inspection.

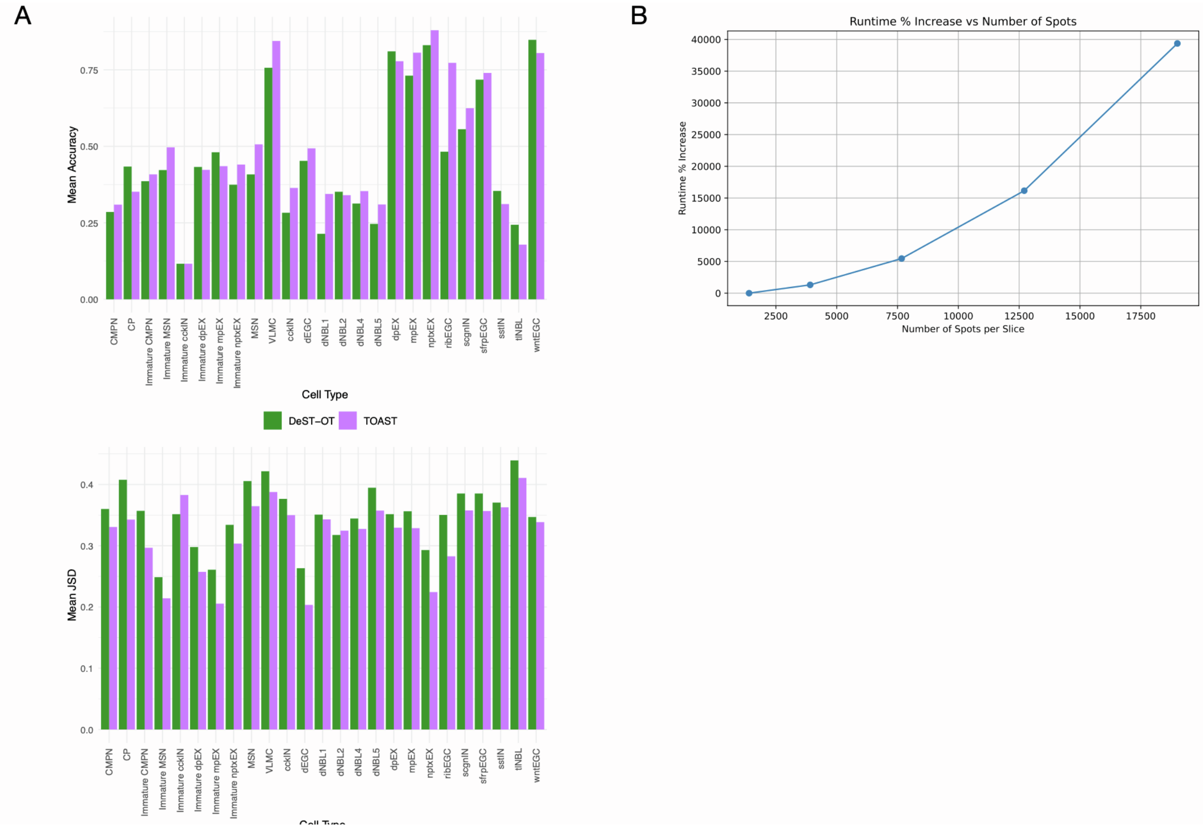

Figure S3: Cell type performance and runtime analysis, related to **Figure 4 and Figure 5**. **A.)** Quantitative comparison of pairwise alignment on Stereo-seq data across all consecutive slices for TOAST and DeST-OT evaluated in terms of accuracy and Jensen-Shannon divergence for each annotated cell type. **B.)** Percentage increase in runtime (seconds) as a function of the number of spots per slice. We simulated two-dimensional spatial transcriptomics datasets with increasing numbers of spatial spots per slice, ranging from 1,500 to 19,000. Consistently with the nature of optimal transport computations, the trend in the plot suggests that the runtime grows approximately with the square of the number of spots, indicating a quadratic time complexity. Empirically, we observed that the runtime remains under one minute for datasets with fewer than 5,000 spots and increases to approximately 7.5 minutes for datasets with nearly 20,000 spots. All timing results were obtained using a single CPU core, without GPU acceleration. To further underscore real-world scalability, we applied TOAST to the integration of scRNA-seq and Visium HD slices[1], confirming that TOAST operates efficiently on high-resolution datasets containing hundreds of thousands of spatial spots.

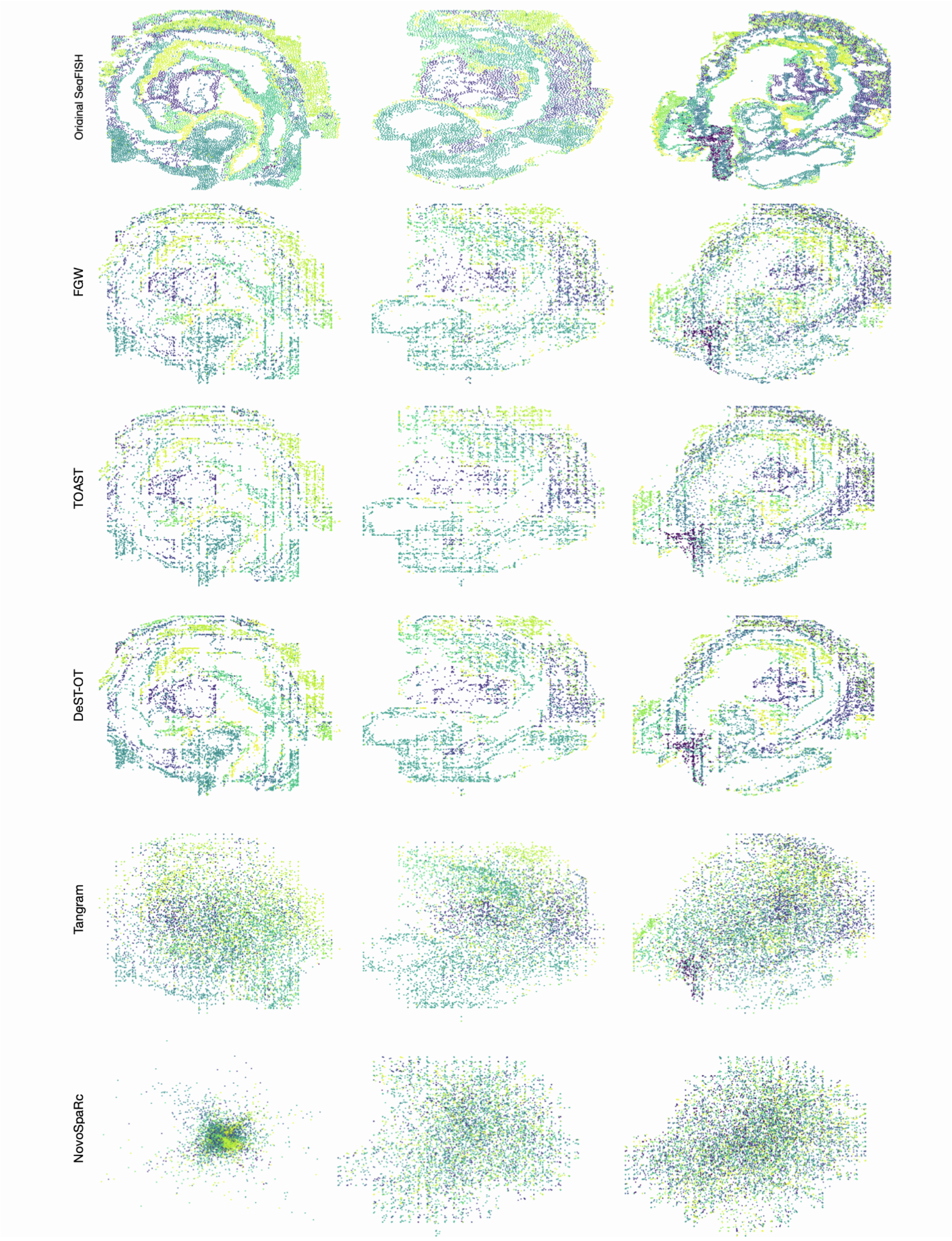

Figure S4: Spatial reconstruction on Mouse Atlas data, related to **Figure 6.** The reconstructed spatial distribution by FWG, TOAST, DeST-OT, Tangram and NovoSparc versus the ground truth spatial distribution. Colors indicate different cell types or regions.

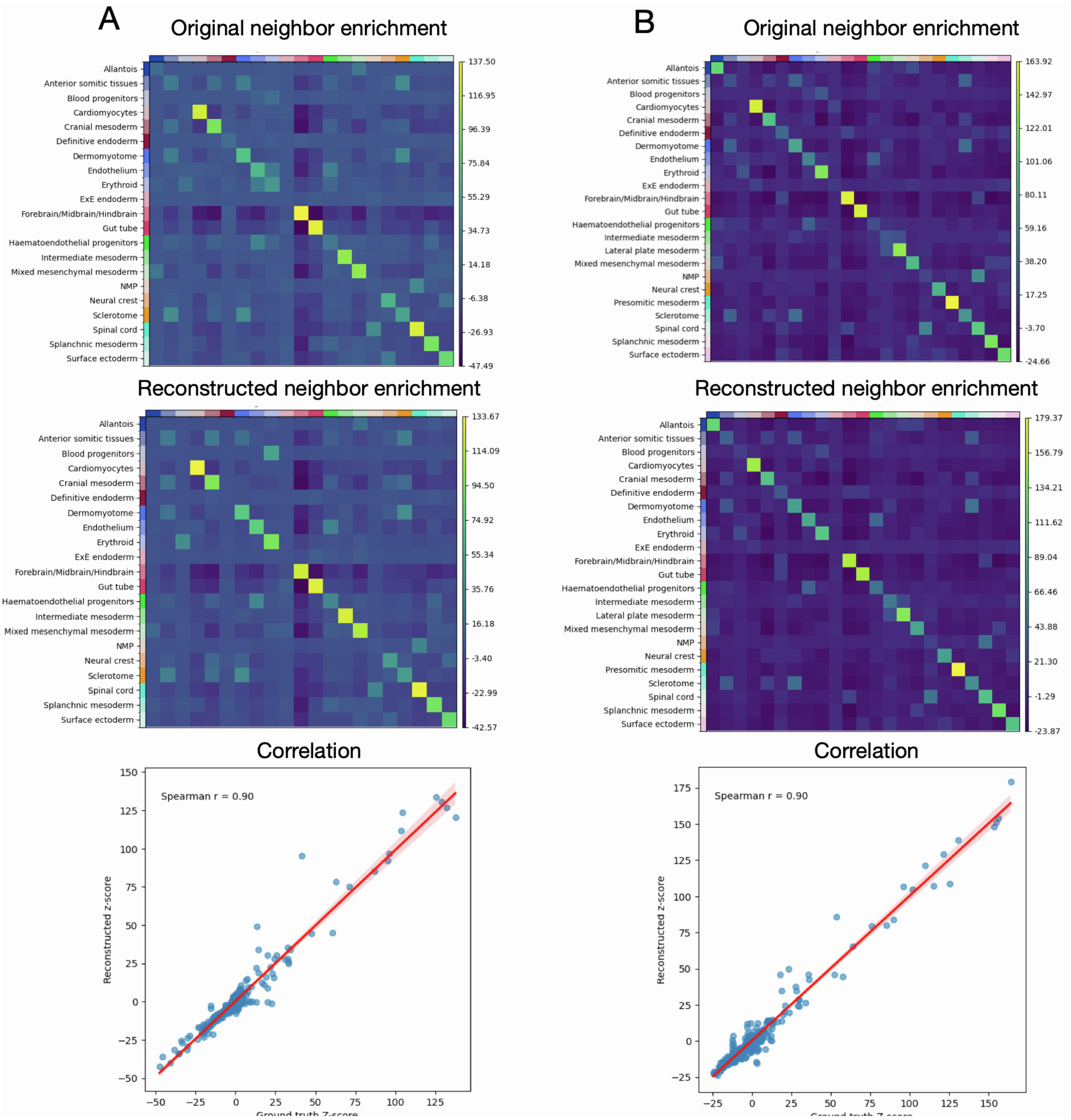

Figure S5: Neighborhood enrichment for individual samples from the Mouse Atlas dataset, related to **Figure 7**. **A.)** Heatmaps showing the neighbor enrichment scores for different cell types in the original slice **(top)**, the reconstructed slice **(middle)**, and correlation between reconstructed and ground truth z-scores **(bottom)** for sample E1z2 and **B.)** E1z3 from the Mouse Atlas.

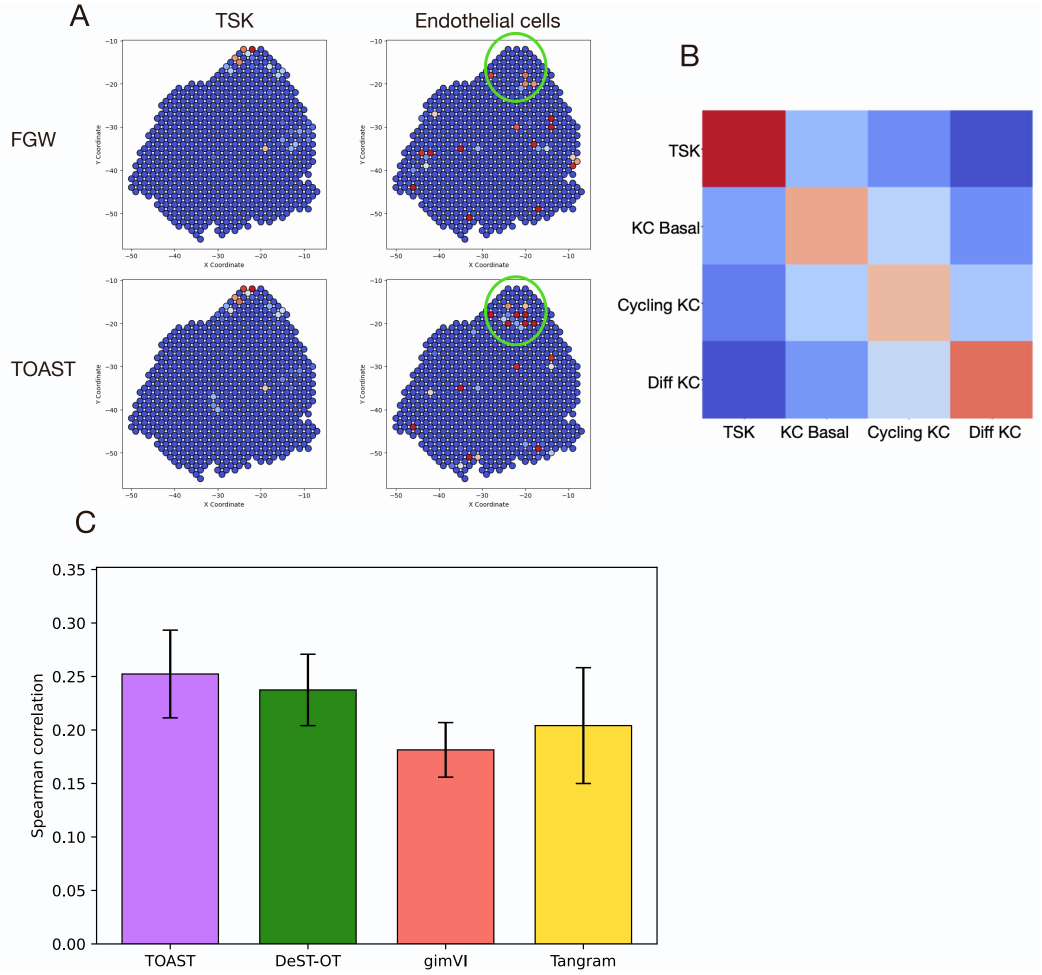

Figure S6: Human squamous cell carcinoma (hSCC) and gene expression imputation, related to **Figure 7**. **A.)** Cell type distribution reconstruction by FGW and TOAST for one donor from the human squamous cell carcinoma (hSCC) dataset[2]. While both FGW and TOAST identified tumor specific keratinocytes, FGW fails to identify the enrichment of endothelial cells closer to the tumor region described in the original publication. **B.)** Neighbor enrichment analysis on tumor keratinocyte subtypes: tumor specific keratinocytes (TSKs) tended to self-aggregate and spatially separate from other KCs. These findings, consistent with those of the original study, are crucial in revealing the spatial characteristics of the tumor microenvironment. **C.)** Comparison of Spearman correlation between predicted and ground-truth spatial gene expression values for held-out genes in the mouse somatosensory cortex dataset across five runs. TOAST achieves higher correlation than DeST-OT, gimVI and Tangram, indicating improved accuracy in unmeasured gene prediction.

References

1. Ciervo, E., Ceccarelli, F., Di Giacomo, A.M., Grisolia, P., Covre, A., Besharat, Z.M., De Falco, A., Caruso, F.P., Laezza, L., Ferraro, L., et al. (2025). Transposable Elements and Homotypic Niches Drive Immune Dynamics and Resistance in Melanoma Epigenetic-based immunotherapy. bioRxiv. https://doi.org/10.1101/2025.10.09.679175.

2. Ji, A.L., Rubin, A.J., Thrane, K., Jiang, S., Reynolds, D.L., Meyers, R.M., Guo, M.G., George, B.M., Mollbrink, A., Bergenstråhle, J., et al. (2020). Multimodal analysis of composition and spatial architecture in human squamous cell carcinoma. Cell *182*, 497–514.
