# Supplementary material for: Topography-aware optimal transport for alignment of spatial omics data
Source: Cell Rep Methods. 2026 Mar 30;6(4):101373. doi: 10.1016/j.crmeth.2026.101373 (PMC13107060; doi:10.1016/j.crmeth.2026.101373)
Supplement: Document S2. Article plus supplemental information [file mmc2.pdf]

Cell Reports
Methods

Article

Topography-aware optimal transport for alignment of spatial omics data

Graphical abstract

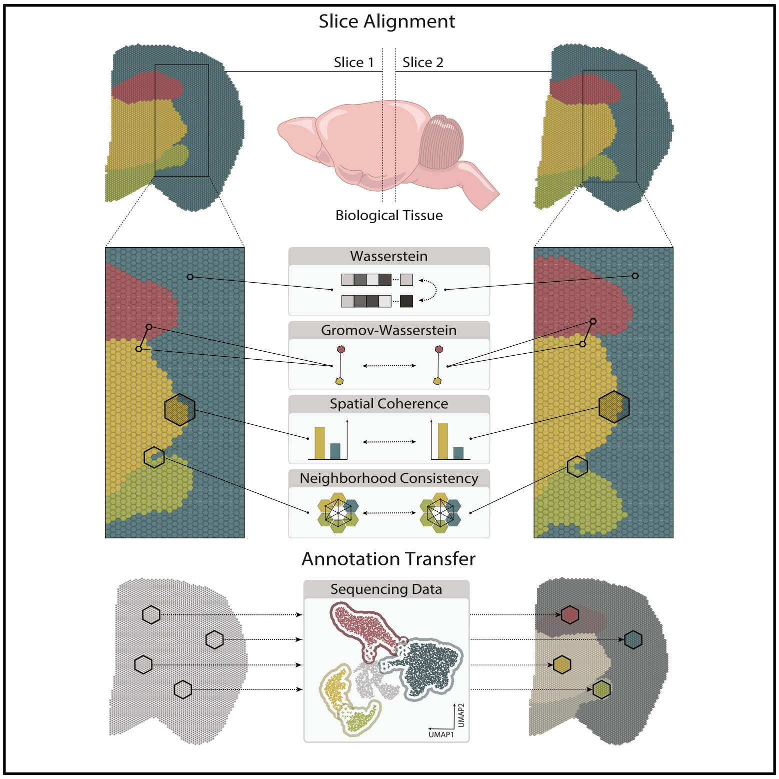

Authors

Francesco Ceccarelli, Pietro Liò, Julio Saez-Rodriguez, Sean B. Holden, Jovan Tanevski

Correspondence

fc485@cam.ac.uk (F.C.),
jovan.tanevski@uni-heidelberg.de (J.T.)

In brief

Ceccarelli et al. introduce TOAST, a topography-aware optimal transport framework for spatial omics alignment. By incorporating spatial coherence and neighborhood consistency, TOAST improves slice alignment, multimodal integration, and spatial reconstruction across diverse transcriptomic and proteomic datasets, enabling biologically interpretable mappings of tissue architecture.

Highlights

- TOAST aligns multimodal spatial omics data
- It integrates spatial constraints into optimal transport
- Preserves local tissue organization and cell-type composition
- Outperforms related OT methods across tasks and datasets

Ceccarelli et al., 2026, Cell Reports Methods *6*, 101373
April 20, 2026 © 2026 The Authors. Published by Elsevier Inc.
https://doi.org/10.1016/j.crmeth.2026.101373

Cell Reports Methods

CellPress
OPEN ACCESS

Article

Topography-aware optimal transport for alignment of spatial omics data

Francesco Ceccarelli,[1,*] Pietro Liò,[1] Julio Saez-Rodriguez,[2,3] Sean B. Holden,[1] and Jovan Tanevski[3,4,5,*]
[1]Department of Computer Science and Technology, University of Cambridge, Cambridge, UK
[2]European Molecular Biology Laboratory, European Bioinformatics Institute (EMBL-EBI), Hinxton, UK
[3]Institute for Computational Biomedicine, Heidelberg University and Heidelberg University Hospital, Heidelberg, Germany
[4]Translational Spatial Profiling Center, Heidelberg University Hospital, Heidelberg, Germany
[5]Lead contact
*Correspondence: fc485@cam.ac.uk (F.C.), jovan.tanevski@uni-heidelberg.de (J.T.)
https://doi.org/10.1016/j.crmeth.2026.101373

MOTIVATION Spatial omics technologies enable high-resolution profiling of gene and protein expression while preserving tissue architecture. A central computational challenge in this context is the alignment of spatially resolved tissue slices across sections, time points, samples, or modalities in order to reconstruct tissue organization, infer developmental trajectories, and integrate multimodal data. Optimal transport (OT), particularly fused Gromov-Wasserstein (FGW), has emerged as a principled framework for spatial alignment by jointly modeling feature similarity and global structural relationships. However, existing OT-based approaches primarily account for global geometry, while neglecting local spatial organization. To address this limitation, we developed TOAST (topography-aware optimal alignment of spatially resolved tissues), a topography-aware extension of FGW that explicitly integrates local spatial constraints into the OT objective. Specifically, TOAST introduces *spatial coherence* and *neighborhood consistency* terms that preserve local heterogeneity and intercellular relationships during alignment. By embedding microenvironmental structure directly into the transport formulation, TOAST produces more accurate, biologically interpretable alignments while remaining computationally scalable.

SUMMARY

Recent advances in spatial omics technologies have provided unprecedented insight into tissue spatial organization, but challenges remain in aligning spatial slices and integrating complementary single-cell and spatial data. Here, we propose TOAST (topography-aware optimal alignment of spatially resolved tissues), an optimal transport (OT)-based framework that extends the classical fused Gromov-Wasserstein (FGW) objective to more comprehensively model the heterogeneity of local molecular interactions. By introducing "spatial coherence," quantified through the entropy of local neighborhoods, and "neighborhood consistency," which preserves the expression profiles of neighboring spots, TOAST's objective function improves the alignment of spatially resolved tissue slices and the mapping between single-cell and spatial data. Through comprehensive evaluations, we demonstrate that our method consistently outperforms traditional FGW and other OT-based alignment methods. By integrating spatial constraints into OT, our framework provides a principled approach to enhance the biological interpretability of spatially resolved omics data and facilitate multimodal data integration.

INTRODUCTION

Single-cell RNA sequencing enables the profiling of a large portion of the transcriptome at the level of individual cells, and it has profoundly advanced our understanding of disease mechanisms and biological processes.[1] Recent advancements in spatial omics technologies, particularly in spatial transcriptomics (ST), enable transcriptome-wide gene expression profiling without dissociation, thereby retaining spatial information and offering a complementary perspective on cellular organization. Experimental protocols for generating ST data are well-established and include, for instance, Visium from 10× Genomics,[2]

This is an open access article under the CC BY license (http://creativecommons.org/licenses/by/4.0/).

GeoMx from NanoString,[3] seqFISH,[4] MERFISH,[5] and Slide-seq2.[6] In the context of ST, the ability to align and compare distributions is critical for studying tissues, where different arrangements of cells dynamically interact to maintain tissue function. Changes in cell composition, structure, and spatial organization often underlie the transition from healthy to diseased states.[7] The alignment of multiple slices is, therefore, critical for improving the generalization of downstream analyses; establishing plausible trajectories in development, disease progression, or response to perturbation; and integrating multimodal spatially resolved omics data.

Optimal transport (OT) has become a foundational tool across fields, including economics, machine learning, and biology.[8–12] By providing a geometry-based approach to realize couplings between two probability distributions, OT facilitates the analysis and alignment of datasets originating from different domains.[13,14] In the context of tissue biology, OT's capacity to match distributions in a principled manner makes it an ideal tool for uncovering cellular relationships and dynamics. Recently, OT has been increasingly adopted for tasks involving data integration and alignment. For instance, PASTE[15] aligns and integrates ST data from multiple adjacent tissue slices, while PASTE2[16] extends PASTE to partially overlapped slices. Similarly, DeST-OT[17] is an OT method for aligning spatiotemporal transcriptomics data, which incorporates cell growth and differentiation objectives in its framework. Related to multislice alignment is the problem of mapping single-cell transcriptomics to spatially resolved imaging/sequencing technologies, hence mitigating the trade-off between lack of spatial information (single-cell technologies) and resolution and gene coverage (spatially resolved technologies). For instance, Rahimi et al.[18] proposed DOT, a multi-objective optimization framework for transferring features across these data modalities, thus integrating their complementary information. In Klein et al.,[19] the authors introduced moscot, a flexible OT framework that implements widely used OT objectives—Wasserstein, Gromov-Wasserstein, and fused Gromov-Wasserstein (FGW)—as well as extensions that relax marginal constraints for unbalanced OT[8] and account for cell growth and death. Collectively, these works highlight the versatility of OT in addressing a broad range of challenges in the integration and alignment of single-cell and spatial omics data, particularly through the use of the FGW objective.

Despite demonstrating promising results, these methods exhibit key limitations that can compromise both their accuracy and biological interpretability. Specifically, while they leverage OT to infer correspondences between slices by accounting for global spatial organization (intra-sample distances), they do not incorporate the local spatial organization, which plays a crucial role in shaping cellular states. In cancer biology, for instance, the tumor microenvironment is highly structured, with immune cells, fibroblasts, and malignant cells forming spatially distinct niches that regulate immune evasion, therapeutic resistance, and tumor progression.[20,21] Similarly, in developmental biology, spatial gradients of morphogens and transcriptional programs orchestrate cellular differentiation and tissue patterning, making local spatial context essential for accurately reconstructing lineage trajectories.[22] Consequently, the omission of domain-specific spatial constraints in OT-based frameworks can lead to biologically implausible mappings that do not capture the true spatial architecture of tissues.

In this work, we introduce TOAST (topography-aware optimal alignment of spatially resolved tissues), a novel topography-aware framework for spatial omics, which explicitly incorporates spatial local constraints into the OT objective. TOAST extends the classical FGW formulation by introducing two additional terms, "spatial coherence" and "neighborhood consistency," to account for spatial organization and molecular heterogeneity. We extensively evaluated TOAST on both simulated and real spatial omics datasets for the tasks of multislice alignment and integration of single-cell and spatial data, demonstrating its advantages over traditional FGW and other alignment methods. Our evaluation is based on data coming from ST technologies such as 10× Visium, Stereo-seq, and seqFISH and spatial proteomics technologies, such as imaging mass cytometry (IMC). By leveraging OT in a spatially informed manner, our method provides a tool for studying tissue organization, tracking cellular dynamics across conditions, and enhancing multimodal data integration.

RESULTS

Topography-aware optimal transport

A spatial omics dataset is composed of a series of spatially resolved tissue slices, each defined by a tuple $\mathcal{S} = (X, S)$ where $X \in N^{n \times p}$ is, for example, in the case of transcriptomics, the matrix of n spots and p genes and $S \in N^{n \times 2}$ contains the spatial coordinates (x,y) of each spot. Given a pair of tissue slices, $\mathcal{S}^1$ and $\mathcal{S}^2$ from two adjacent tissue sections or time points containing n_1 and n_2 spots, respectively. OT aims to find a transport matrix $\Pi_+ \in R^{n_1 \times n_2}$, whose positive entry Π_{ij} describes the probability of transporting spot i in $\mathcal{S}^1$ to spot j in $\mathcal{S}^2$. To this end, several recent works[15,16,19] proposed the use of the FGW distance and minimized the following objective function:

$$\Gamma(\Pi) = (1 - \alpha)\langle \Pi, M \rangle_F + \alpha \sum_{i,j,k,l} L\left(C_{1,i,k}, C_{2,j,l}\right)\Pi_{i,j}\Pi_{k,l}. \quad \text{(Equation 1)}$$

The Frobenius inner product term, $\langle \boldsymbol{\Pi}, \boldsymbol{M} \rangle_F$, corresponds to the *Wasserstein component* as it minimizes the pairwise transport cost encoded in the cost matrix $\boldsymbol{M}$. In the context of ST, the Wasserstein term encourages mappings between spots with similar expression profiles (STAR Methods, section distance functions). The second term, $\sum_{i,j,k,l} L\left(C_{1,i,k}, C_{2,j,l}\right)\boldsymbol{\Pi}_{i,j}\boldsymbol{\Pi}_{k,l}$, represents the Gromov-Wasserstein component, as it captures the structural dissimilarity between spatial slices. $C_{1,i,k}$ and $C_{2,j,l}$ represent the intra-slice spatial distance between spots i and k and spots j and l in $\mathcal{S}^1$ and $\mathcal{S}^2$, respectively. Here $L(C_{1,i,k}, C_{2,j,l})$ measures the dissimilarity between the local contexts C_1 and C_2, making the OT framework sensitive to relational or structural differences (STAR Methods, section distance functions). The hyperparameter $\alpha \in [0,1]$ controls

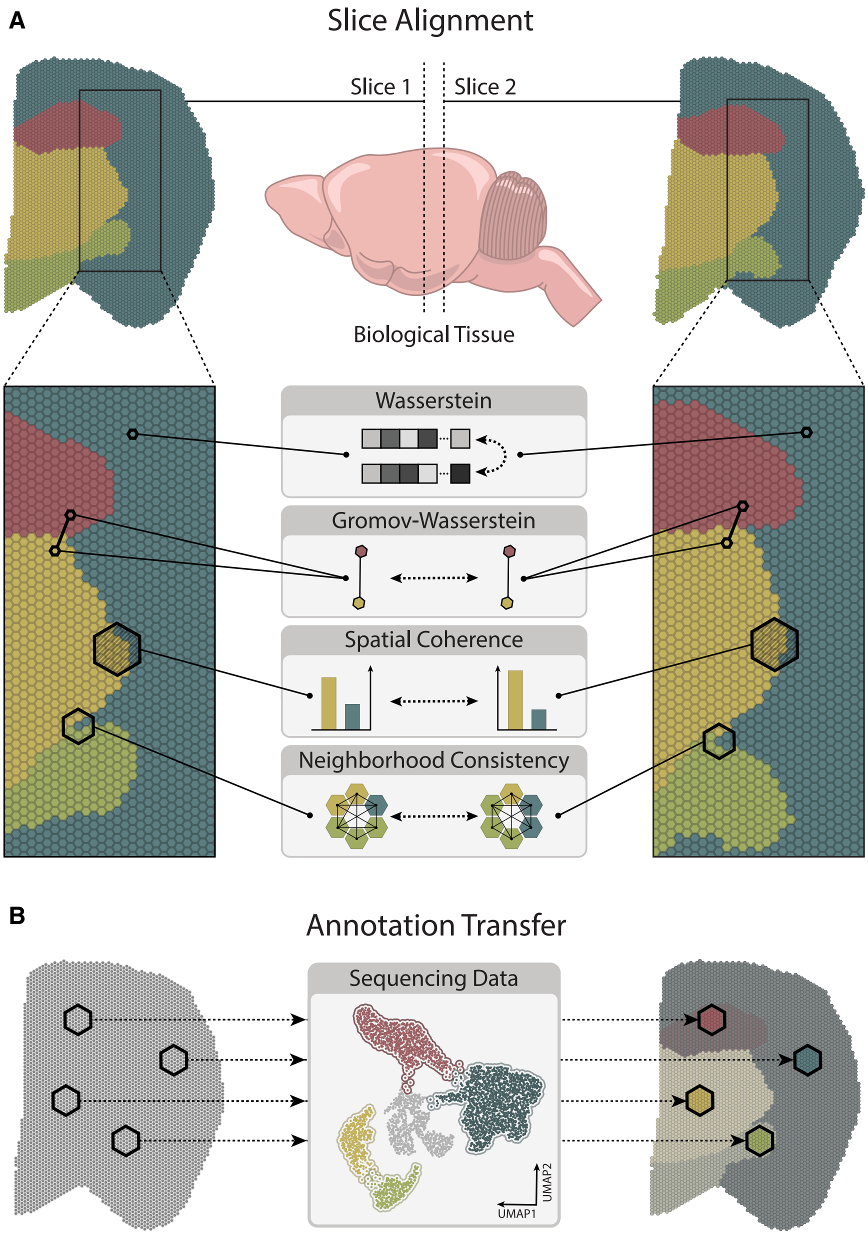

Figure 1. TOAST: A topography-aware fused Gromov-Wasserstein optimal transport framework for spatial omics

(A) Slice alignment: we align spatial tissue slices by incorporating spatial coherence and neighborhood consistency into the optimal transport objective to better preserve local tissue organization and molecular heterogeneity.

(B) Annotation transfer: TOAST enables the integration of multiple slices of spatially resolved omics as well as localization of dissociated cells to spatial locations by mapping cellular states while preserving the spatial context.

the balance between the Wasserstein and Gromov-Wasserstein components.

The state and function of cells within a tissue are affected by interactions with neighboring cells, extracellular matrix components, and local signaling gradients.[23] The intra- and intercellular relationships form distinguishing and representative spatial patterns across tissues and conditions.[24,25] Processes such as immune responses, tissue regeneration, and cancer progression often depend on the spatial organization and interaction of different cell types. Furthermore, recent studies on tissue organization in cancer patients have identified the presence of spatially clustered regions, dominated by a single-cell state, and disorganized regions, exhibiting a variety of cell states.[26] As a result, it is pivotal to be able to model these interactions and account for molecular heterogeneity while defining a successful transport plan. To this end, we introduce TOAST, a generalized topography-aware OT-based framework for intra- and inter-sample and temporal alignment and mapping of spatially resolved omics data (Figure 1). To decouple the diversity of cell states from the aggregated expression in the immediate neighborhood of each cell, TOAST incorporates two new terms to the objective function in Equation 1.

First, we introduce a spatial coherence term, designed to capture the heterogeneity in the spatial neighborhood of each spot. In detail, using the (x,y) coordinates, we construct a spatial graph G where each node represents a spot and edges connect each spot to its closest neighbors. Given a node i, we define $P_i(k)$ to be the probability of a neighbor of i belonging to class $k \in K$, where K denotes the set of cell labels. We then compute the spatial coherence score for a node i as the entropy of the label distribution around i:

$$H(i) = -\sum_{k \in K} P_i(k) log(P_i(k)). \quad \text{(Equation 2)}$$

The cell labels typically correspond to manually annotated cell types or cell states or labels transferred from an available single-cell reference atlas. In the context of the optimization task, however, annotations are not strictly required. Instead, a set of discrete cell labels can be obtained by clustering highly variable genes across tissue slices. $H(i)$ provides a straightforward interpretation: it quantifies the degree of heterogeneity of a given spot's neighborhood. A higher $H(i)$ value indicates a more diverse neighborhood, with multiple cell labels being represented among the neighboring spots. Conversely, a lower $H(i)$ value suggests a more homogeneous neighborhood,

dominated by a single-cell label. Incorporating spatial coherence into the OT objective function enables a more refined modeling of spatial interactions. Specifically, it encourages solutions that respect the local heterogeneity of the tissue environment, ensuring that the transport plan aligns with the tissue's local structure. Following the notation in Equation 1, we define C_3 to be the dissimilarity of the spatial entropy scores of spot i in $\mathcal{S}^1$ and spot j in $\mathcal{S}^2$ as

$$\mathbf{C_3}(i,j) = L(H(i), H(j)). \quad \text{(Equation 3)}$$

Second, to further enhance the preservation of the spatial context and model the locally consistent intercellular relationship patterns,[24,25] we introduce a neighborhood consistency term. This additional term is designed to ensure that the average expression profiles of a cell's neighborhood are preserved across slices. As before, a graph G is constructed using the (x,y) spatial coordinates. For each node i in G, we consider its set of neighbors $\mathcal{N}(i)$ and compute the average expression profile across all genes within this neighborhood. We define C_4 to be the distance between the average neighborhood expression profiles of spot i in $\mathcal{S}^1$ and spot j in $\mathcal{S}^2$ as

$$\mathbf{C_4}(i,j) = L\left(\overline{E}_{\mathcal{N}(i)}, \overline{E}_{\mathcal{N}(j)}\right), \quad \text{(Equation 4)}$$

where $\overline{E}_{\mathcal{N}(i)}$ and $\overline{E}_{\mathcal{N}(j)}$ represent the average expression profiles of the neighborhoods of spots i and j, respectively. The neighborhood consistency acts as a regularization term and penalizes transport plans that map spots exhibiting neighborhoods with drastically different expression profiles, thereby ensuring that the spatial context of each spot is preserved across slices.

By integrating both the spatial coherence and neighborhood consistency terms into the classical FGW formulation, we define the objective function of TOAST as

$$\Gamma(\Pi) = (1-\alpha)\langle \Pi, M\rangle_F + \frac{\alpha}{3}\left[\sum_{i,j,k,l} L\left(C_{1,i,k}, C_{2,j,l}\right)\Pi_{i,j}\Pi_{k,l} + \langle \Pi, C_3\rangle_F + \langle \Pi, C_4\rangle_F\right], \quad \text{(Equation 5)}$$

where $\alpha \in [0,1]$ controls the trade-off between the feature similarity term and the equally weighted structural terms. In practice, an entropy-based regularization term $H(\mathbf{\Pi}) = \sum_{i,j}\mathbf{\Pi}_{i,j}\log\left(\mathbf{\Pi}_{i,j}\right)$ is added to the optimization problem in Equation 5 to control for smoothness and accelerate the optimization of Π.[27] We further constrain the optimization such that the total mass transported from $\mathcal{S}^1$ exactly matches the total mass transported to $\mathcal{S}^2$ resulting in the balanced OT optimization formulation

$$\min \Gamma(\boldsymbol{\Pi}) - \varepsilon H(\boldsymbol{\Pi}), \quad \text{(Equation 6)}$$

subject to:

$$\boldsymbol{\Pi} 1_{n_2} = \boldsymbol{u}, \boldsymbol{\Pi}^\top 1_{n_1} = \boldsymbol{v}, \boldsymbol{\Pi} \geq 0,$$

where $H(\Pi)$ is the entropy of the transport matrix, $\varepsilon > 0$ controls the strength of this entropic regularization, $u \in R^{n_1}$ and $v \in R^{n_2}$ are the mass vectors corresponding to the distributions of spots in $\mathcal{S}^1$ and $\mathcal{S}^2$, and 1_n is a vector of ones.

Evaluation on simulated data

One-dimensional simulation

Following the evaluation scenario presented by Halmos et al.,[17] we simulated one- and two-dimensional slices with eight-dimensional feature vectors for each spot (STAR Methods, section "one-dimensional simulation"). For the one-dimensional simulation, we defined two tissue slices $\mathcal{S}^1$ and $\mathcal{S}^2$, each containing 101 spots and two different cell types, A and B. The feature vectors for each cell type were designed to be orthogonal and dependent on the one-dimensional spatial location of each spot.[17] $\mathcal{S}^1$ contained 51 spots of cell type A and 50 spots of cell type B, while $\mathcal{S}^2$ had 49 spots of cell type A and 52 of cell type B. In addition, to mimic the organization of real biological tissues, we introduced clustered regions, where only one cell type was present, and disorganized regions, where both cell types were mixed. Figure 2A shows an example of the spatial organization of the simulated one-dimensional ST data.

We compared the classical FGW formulation in Equation 1, with our topography-aware formulation, TOAST, which integrates spatial coherence and neighborhood consistency in the objective function as in Equation 5. As evaluation metric, we assessed mapping accuracy as the sum of probabilistic alignment weights Π_{ij} over all pairs (i,j) of spots in the true alignment. For the true alignment, spots were defined as corresponding if they shared the same cell type or were located in regions with the same spatial organization (that is, clustered or disorganized). We defined the former as "cell type accuracy" and the latter as "coherence accuracy." For our experiments, we tested multiple α values (0.1, 0.3, 0.5, 0.8, 1), progressively increasing the weight of the spatial component. Figure 2B shows the comparison between FGW and TOAST in terms of these metrics. TOAST consistently matches or outperforms FGW in terms of cell type accuracy across all α values, indicating its robustness in preserving cell identity. Compared to FGW, our topography-aware transport plan preserves the spatial neighborhoods of the slices, as reflected by the coherence accuracy metric.

Figure 2C shows a qualitative comparison between the transport maps from FGW and TOAST. In TOAST's alignment, connections in disorganized regions are notably stronger and better mapped, reflecting the model's ability to preserve the local context of spatially disorganized areas. In contrast, FGW yields weaker and less-consistent connections, as it primarily minimizes transport and structural costs without accounting for the molecular heterogeneity or the neighborhood context of the cells. To dissect the contribution of each spatial constraint within the TOAST objective, we conducted an ablation analysis by systematically removing either the spatial coherence or neighborhood consistency term. In Figure S1, we show that the spatial coherence term primarily supports the accurate mapping of cell types, while the neighborhood consistency term stabilizes the alignment by encouraging cells in well-defined tissue domains to map together. The full TOAST model achieves the highest scores across both metrics, demonstrating that the two terms provide complementary, non-redundant information.

Two-dimensional simulation

Similar to the one-dimensional case, we simulated ST data in two dimensions (STAR Methods, section two-dimensional simulation). Both slices contained 624 spots, with $\mathcal{S}^1$ being composed

CellPress
OPEN ACCESS

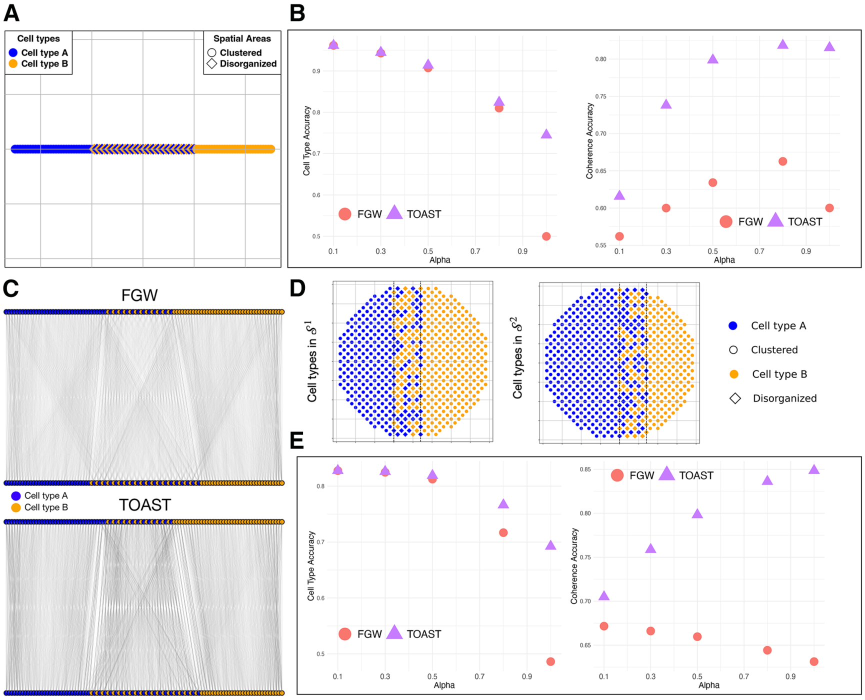

Figure 2. Evaluation on one- and two-dimensional simulated spatial slices

(A) Example of the spatial cell type distribution for slice $\mathcal{S}^1$ for the one-dimensional simulation.

(B) Accuracy comparison between the traditional FGW formulation and our topography-aware formulation, TOAST, in terms of cell type and spatial coherence for the one-dimensional simulated ST data.

(C) Transport maps computed by FGW (top) and TOAST (bottom). Edges represent transport probabilities between source and target cells; only connections with transport weight $\geq 1 \times 10^{-5}$ are shown. Edge intensity and thickness are proportional to the transport mass, allowing visual comparison of strong versus weak couplings.

(D) Spatial cell type distributions for slice $\mathcal{S}^1$ and $\mathcal{S}^2$ for the two-dimensional case.

(E) Accuracy comparison between FGW and TOAST in terms of cell type and spatial coherence for the two-dimensional simulated ST data.

of 278 spots of cell type A and 346 spots of cell type B and $\mathcal{S}^2$ of 385 spots of cell type A and 239 spots of cell type B. As in the one-dimensional case, we defined clustered and disorganized areas (Figure 2D). Figure 2E shows the transport map accuracy for the two-dimensional case, comparing FGW and TOAST across different values of α. On both cell type and coherence accuracy, TOAST consistently outperforms the traditional FGW formulation. For small values of α, the cell type mapping performance remains similar as the spatial information is not fully exploited. As α increases, the performance of FGW declines markedly compared to TOAST. At higher α values, FGW fails to correctly map cells to spatial regions, whereas TOAST better captures local context and preserves spatial coherence.

Intra- and intersample and temporal alignment

Human dorsolateral prefrontal cortex Visium

To assess the ability of our framework to map spots across slices in a real dataset, we analyzed a human dorsolateral prefrontal cortex (DLPFC) data generated using 10× Visium.[2] This study sequenced 12 tissue slices spanning six neuronal layers plus white matter from the DLPFC in three human brains. Manual annotations of the tissue layers were provided in the original study. One of the samples from this dataset is shown in Figure 3A.

We assessed the pairwise alignment of all consecutive slices for each sample and compared the accuracy, measured by the number of correctly matched cell annotation pairs across the slices, between FWG (Equation 1), TOAST (Equation 5), DeST-OT,[17] Tangram,[28] Paste2,[15,16] and NovoSparc.[29] Figure 3B presents the results of this comparison. By explicitly modeling neighborhood composition and spatial expression, TOAST outperforms the other methods across all pairwise alignments and samples. TOAST's superior performance stems from its ability to explicitly model local cell type composition within the tissue. Given that the brain cortex is a highly layered and spatially organized structure (Figure 3A), where distinct neuronal and glial cell populations are arranged in well-defined regions, a transport plan incorporating neighborhood consistency and spatial coherence is able to yield more biologically meaningful mappings. By preserving the local spatial organization of spots, TOAST ensures that cell type transitions align with the inherent structural properties of the cortex, leading to more accurate cross-slice alignment. While DeST-OT and FGW also show good accuracy, Tangram and NovoSparc fail to align spatial slices accurately in most comparisons, leading to the lowest scores in Figure 3B. Unsurprisingly, Paste2 reaches similar results to FGW, as they share the same objective function.

We further explored the ability of the models to reconstruct the local spatial heterogeneity by comparing the cell type distributions between cells in the source slice and cells in the aligned slice by quantifying the Jensen-Shannon divergence (JSD) of their nearest 20 neighbors. A lower JSD value signifies a closer resemblance in cell type distribution between predicted and true cell neighbors. Figure 3C shows that TOAST better preserves the spatial cell type composition compared to other methods, consistently reaching lower JSD scores. Furthermore, even when the coherence term is defined using unsupervised cell labels derived from Leiden clustering,[30] TOAST still exhibits the strongest performance (Figure S2A).

To further demonstrate the advantages of TOAST, we show that the alignment matrices between consecutive slices can be successfully used to reconstruct a coherent three-dimensional tissue architecture (Figure S2B). By sequentially applying the learned transport plans, we propagate spatial coordinates across neighboring slices and recover the overall volumetric organization of the tissue.

Along with alignment of consecutive slices, we designed a more challenging set of experiments involving alignment of

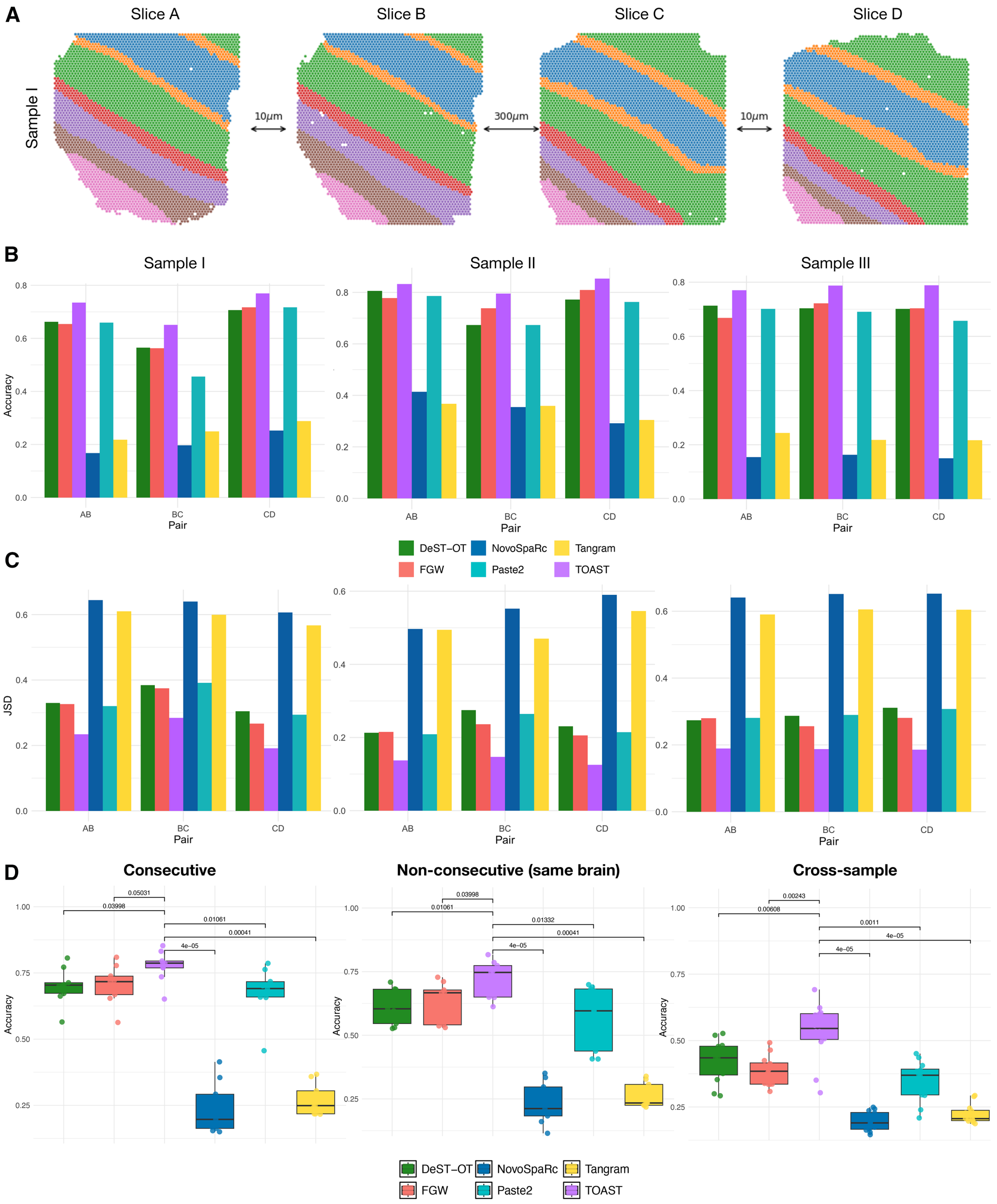

Figure 3. Evaluation on the human DLPFC data

(A) One sample of human dorsolateral prefrontal cortex (DLPFC) with four slices where spots are colored according to the original annotations.

(B and C) Quantitative comparison of the pairwise alignment of all consecutive slices for FWG, TOAST, DeST-OT, Tangram, Paste2, and NovoSparc in terms of accuracy and JSD, respectively.

(D) Accuracy results for all methods on consecutive slices, non-consecutive slices from the same brain, and cross-sample alignments. *p* values were computed using two-sided Wilcoxon rank-sum tests.

non-consecutive slices from the same brain and cross-sample alignment (a slice from one sample is pairwise aligned with a slice from another sample). Figure 3D shows the accuracy results of this comparison. As in the consecutive alignment experiments, Tangram and NovoSparc achieve the lowest accuracy, with DeST-OT and FGW exhibiting higher accuracy scores. However, as illustrated in the boxplots and measured by the Wilcoxon rank-sum test, TOAST outperforms all the other methods with

a statistically significant margin across all three experiments. Finally, our topography-aware framework also exhibits the strongest performances when comparing the average accuracy and JSD for each cell annotation across all three experiments (Figure S2C–S2E). To further illustrate the biological interpretability of TOAST outputs, we quantified how well each method preserved local tissue structure using spatial coherence scores. As shown in Figure S2F (upper panel), TOAST maintains the original distribution of spatial coherence more faithfully than FGW, indicating better preservation of microenvironmental organization. We additionally computed alignment confidence scores (Figure S2F, lower panel), defined as the maximum transport mass assigned to each cell. These confidence maps facilitate the identification of reliably aligned regions, as well as transitional or heterogeneous zones that might merit further biological investigation.

Axolotl brain Stereo-seq

In Wei et al.,[22] Stereo-seq was used to measure gene expression in the telencephalon, a brain region in the axolotl (*Ambystoma mexicanum*), a species of salamander, at different developmental time points. Figure 4A depicts the spatial organization of cells across the five developmental stages (stage 44, stage 54, state 57, juvenile, and adult) colored by cell type.

We assessed the pairwise alignment of all consecutive slices and compared TOAST against the best-performing method from the previous section, DeST-OT. Figure 4B shows that across all values of α, by explicitly taking into account spatial neighborhood information, TOAST consistently reaches the highest accuracy. Although the improvement margin is smaller than in the well-layered human DLPFC, TOAST is still capable of outperforming DeST-OT in all alignments.

In Figure 4C, we quantify the models' ability to preserve cell type composition across alignments. TOAST exhibits lower JSD scores in all pairwise alignments, indicating that it better preserves the composition of the local spatial neighborhood compared to DeST-OT, which shows greater deviation from the original cell type distributions (performances for each individual cell type are shown in Figure S3A). We then examined the cell type transition matrix produced by TOAST for each pair of consecutive time points. We depict the most frequent transitions, supported by at least 50 occurrences, in Figure 4D.

Consistent with findings in Halmos et al.,[17] we observed that immature cell types expressing early developmental markers disappear after the juvenile stage, where immature cell types like common myeloid progenitor or medium spiny neurons (MSN) transition into their respective mature cell types. Developmental ependymoglial cells (dEGCs) disappear at stage 57, with TOAST predicting their primary transition into ribEGCs, which reside in the ventricular zone—the same spatial region as dEGCs. Finally, a directed cycle between cckIN and MSN emerges, particularly during the juvenile-to-adult transition, likely due to their co-localization in the striatum region of the brain.

Applicability of TOAST beyond ST

While all experiments so far focused on transcriptomics data, our OT framework finds application also on other types of spatial omics. As an example, we considered IMC from sections of 4 patients with squamous cell carcinoma of head and neck, breast cancer, non-small cell lung cancer, and colorectal cancer, respectively, from the Integrated Immunoprofiling of Large Adaptive Cancer patient cohorts[31] (IMMUcan). Single-cell measurements for 40 proteins, along with annotations of major immune and cancer cell types, were available for each section.

Figure 5A shows four sections, annotated by the cell type and by the expression of three markers (CD8, CD68, and CDH1). We aligned all intra-sample slices, resulting in a total of 18 comparisons, and used the cell type annotations to measure the accuracy of DeST-OT and our topography-aware formulation, TOAST. Figure 5B quantifies the accuracy of both methods across all alignments. The results indicate that TOAST consistently outperforms DeST-OT across all patients, demonstrating its superior ability to establish biologically meaningful correspondences between aligned slices. TOAST also achieves higher accuracy for all but one cell type compared to DeST-OT (Figure 5C). Given the computational demands of large-scale spatial omics analyses, Figure 5D shows the execution time distributions for both methods. These results, along with the findings in Figure S3B, where we performed a controlled runtime analysis using simulated datasets of increasing size, indicate that TOAST not only improves alignment accuracy but also remains tractable even at tens of thousands of spatial locations, making it a scalable and efficient approach for spatial omics data integration.

To evaluate the performance of TOAST in a multimodal setting, we applied it to integrate ST and spatial proteomics measured on the same formalin-fixed paraffin-embedded renal carcinoma sample from the Xenium platform[32] (Figure 5E). From the multimodal measurements, we constructed two matched spatial omics views: (1) transcript-level expression for the 405 genes in the base Xenium panel and (2) protein expression for the 25 immunophenotyping targets included in the protein panel. In this way, we simulate a scenario where different modalities are measured on consecutive slices with a small panel overlap, while still having ground-truth locations available for evaluation. For each modality, we performed independent preprocessing and unsupervised Leiden clustering to obtain label-free partitions required for the spatial coherence term in TOAST. We then aligned the two modalities using both classical FGW and DeST-OT and our spatially informed variant, TOAST. Because both assays were derived from the same physical cells, the ground-truth correspondence between transcript and protein measurements is known. We, therefore, quantified alignment quality using true one-to-one cell matches, allowing a direct and biologically meaningful assessment of each method's ability to recover the correct multimodal mapping. Figure 5F shows that TOAST achieves higher alignment accuracy compared to both FGW and DeST-OT, demonstrating that the additional spatial terms improve multimodal correspondence between transcript and protein maps.

Spatial reconstruction

Even though our OT methodology is not designed for the task of mapping spatial locations to single-cell data, we can apply it under the assumption of regional association of the gradient of expression to the spatial location, which can be best observed in the context of early development.[33]

To this end, we performed semi-simulation experiments on the synthetic data generated from the Spatial Mouse Atlas

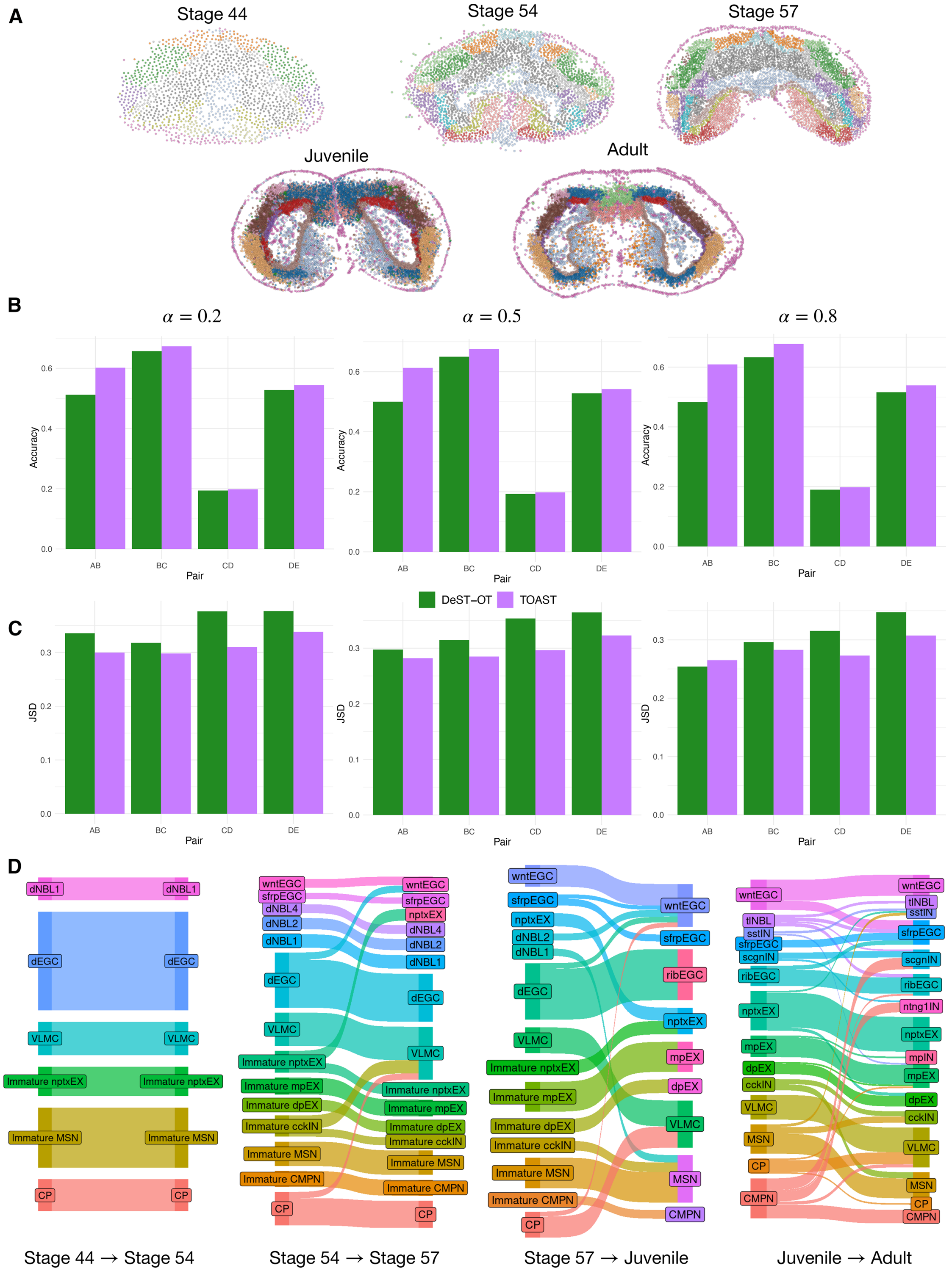

Figure 4. TOAST recovers developmental trajectories in Stereo-seq axolotl brain

(A) Stereo-seq at different developmental time points colored by cell types.

(B and C) Quantitative comparison of the pairwise alignment of consecutive slices for TOAST and DeST-OT in terms of accuracy and JSD, respectively.

(D) Most frequent cell type transitions predicted by TOAST for each pair of consecutive developmental stages. Our framework accurately captures the development of immature cells into their respective mature cell types.

CellPress
OPEN ACCESS

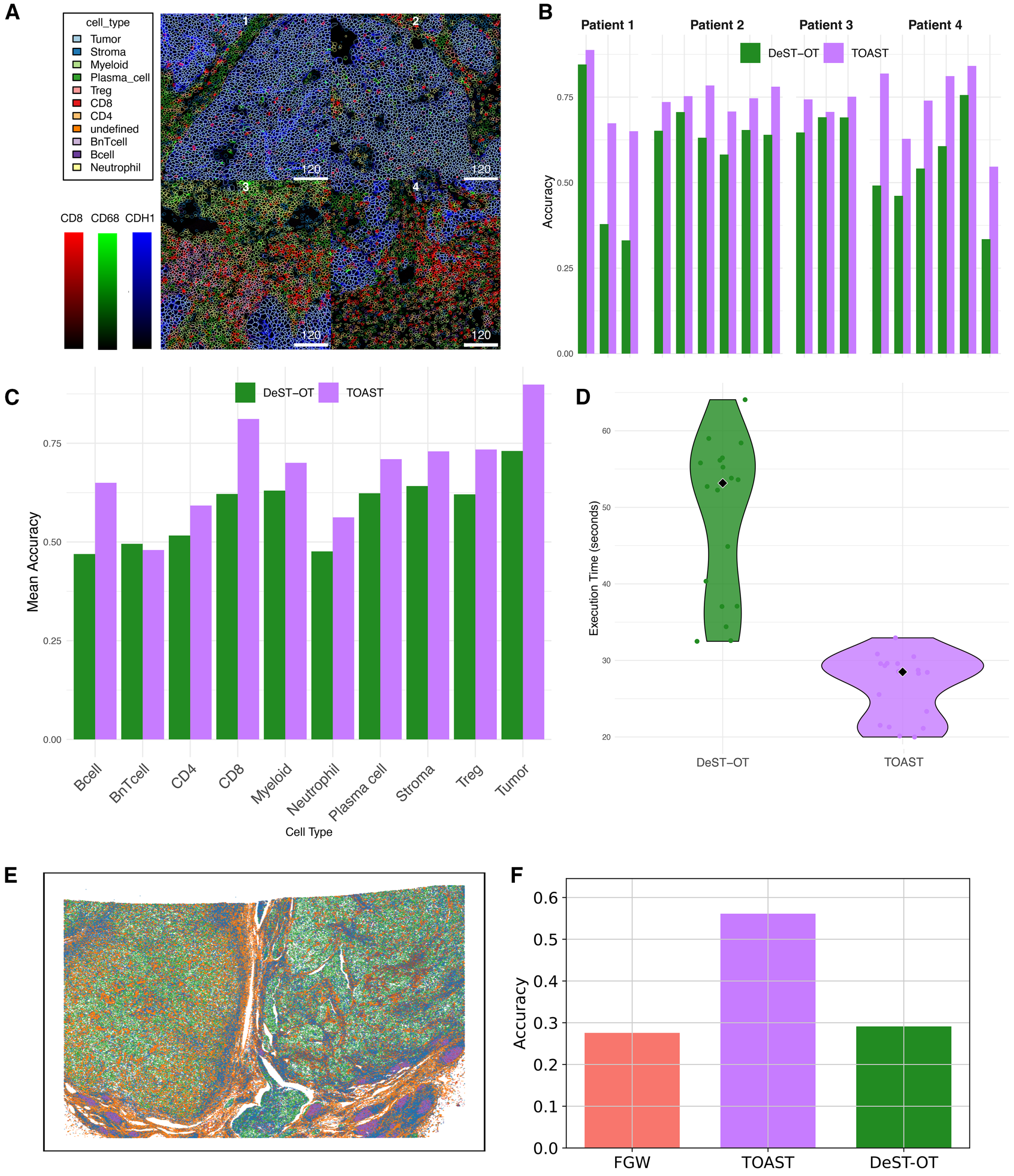

Figure 5. TOAST accurately aligns different types of spatial omics

(A) For four example sections from IMMUcan, the spatial distribution of different cell types is shown, with colors representing CD8a, CD68, and CDH1 protein expression. The outlined cells correspond to different annotated cell types.
(B) Accuracy for TOAST and DeST-OT for all intra-sample slice alignments. Patients 1 and 3 and Patients 2 and 4 have 3 and 4 sections, respectively.
(C) Mean accuracy for TOAST and DeST-OT across different cell types.
(D) Execution time distribution for DeST-OT and TOAST. The violin plots illustrate the variability in computation time, while the black diamond markers indicate the median execution time, in seconds, for each method.
(E) Renal carcinoma sample from the Xenium platform.
(F) Accuracy comparison of FGW, DeST-OT, and TOAST using ground-truth correspondence between transcript and protein measurements.

datasets.[34] Because this dataset provides experimentally measured cell coordinates, this experiment is designed as a controlled reconstruction task that enables quantitative benchmarking against ground-truth spatial locations, rather than a fully unpaired single-cell-to-spatial integration scenario where such true spatial positions would otherwise be unavailable.

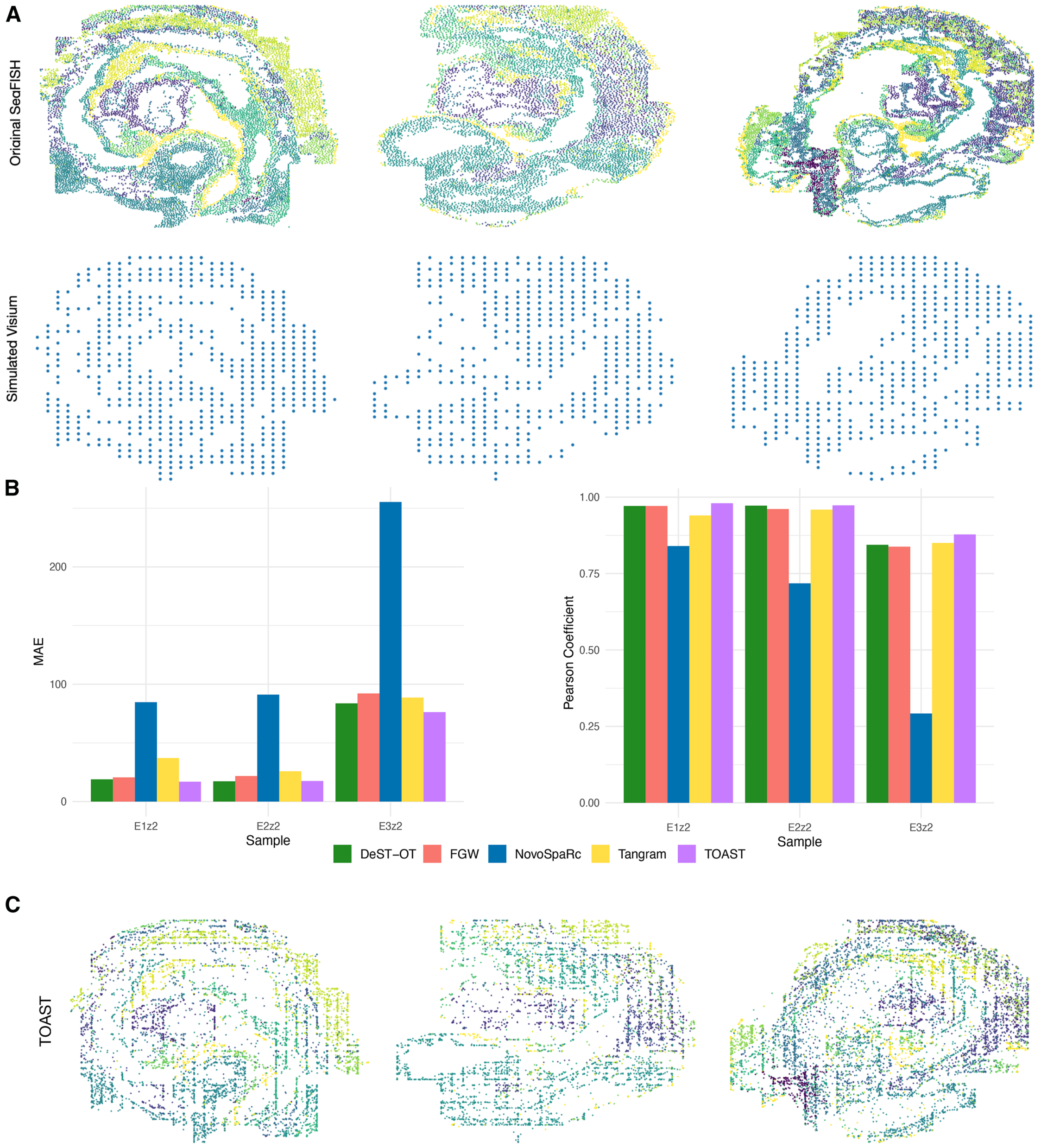

Figure 6. TOAST reconstructs spatial relationships in semi-simulated spatial transcriptomics data

(A) Original seqFISH spatial data and simulated 10× Visium with lower resolution and coverage.

(B) MAE and Pearson correlation between ground truth and predicted locations for three embryo slides for FWG, TOAST, DeST-OT, Tangram, and NovoSparc.

(C) The reconstructed spatial distribution by TOAST versus the ground-truth spatial distribution. Colors indicate different cell types or regions.

Three different embryo slides were profiled with seqFISH,[4] and single-cell gene expression profiles were provided along with the spatial coordinates of the cells. Following the approach in Hao et al.,[35] we treated the gene expression data as pseudo-single cell data without the spatial coordinates and treated the spatial coordinates as the ground truth to test the predictions of our transport plan. We generated pseudo-ST data to mimic key characteristics of the widely used 10× Visium platform[2] lower-than-single-cell resolution and partial coverage of cells within the tissue (Figure 6A). The gene expression for each spot was calculated as the sum of the gene expression values of all the covered cells, while the true cell type proportions for each spot were determined based on the cell type annotations of the cells it encompassed.

We compared TOAST to traditional FGW, DeST-OT, NovoSpaRc, and Tangram. Given a transport plan obtained by any of the aforementioned methods, the spatial coordinates of a single cell were computed as the average of the coordinates of the Visium spots mapped to that cell, weighted by the transport probabilities. Mean absolute error (MAE) and Pearson

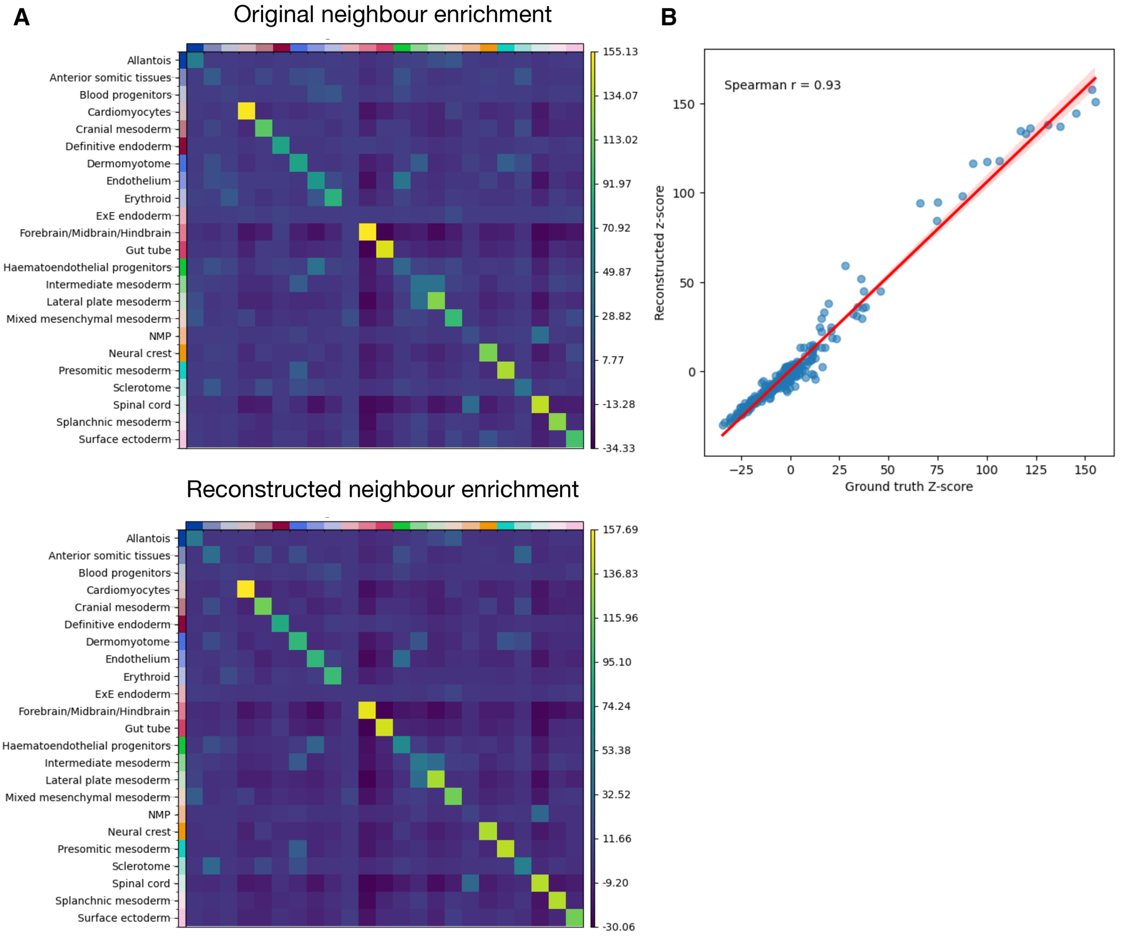

Figure 7. Spatial reconstruction performance of our topography-aware OT framework on one sample from the Spatial Mouse Atlas

(A) Comparison of original and reconstructed neighbor enrichment for sample E1z1 from the Spatial Mouse Atlas. Heatmaps show the neighbor enrichment scores for different cell types in the original slice (top) and the reconstructed slice (bottom). The TOAST-reconstructed enrichment matrix closely mirrors the original, demonstrating the model's ability to preserve local tissue architecture.

(B) The strong correlation between reconstructed and ground truth Z scores confirms the similarity between the original and reconstructed spatial interactions.

correlation coefficient were used to measure the distance between the ground truth and predicted locations. Figure 6B shows the results of this comparison.

On both metrics, TOAST consistently outperforms the other methods, with DeST-OT and FGW reaching slightly lower performances. Tangram and NovoSpaRc show significantly worse spatial reconstruction abilities compared to the other methods. For each of three embryo slides, in Figure 6C, we show that the transport matrix produced by TOAST learns a mapping that strongly preserves the topology of the original single-cell space. Figure S4 shows this qualitative comparison for all the evaluated methods. As quantitatively indicated in Figure 6, FGW, DeST-OT, and TOAST are better able to reconstruct the spatial relationships compared to Tangram and NovoSpaRc, which fail to preserve the spatial distribution of the single cells.

Next, we assessed the ability of TOAST to reconstruct the cell type relationships by computing a proximity-based enrichment score (STAR Methods, section neighborhood enrichment) for each cell type in the original and reconstructed seqFISH data. For one of the samples, Figure 7A shows that the reconstructed spatial cell type relationships closely resemble the ones in the original data. A high Spearman coefficient, Figure 7B, confirms this similarity. Figures S5A and S5B depict the same analysis and similar results for the remaining samples.

We performed a similar qualitative evaluation of our framework to analyze the tumor microenvironment in a human squamous cell carcinoma dataset.[36] Figures S6A and S6B show that TOAST is able to accurately reconstruct the cell type composition and the spatial relationships of the tumor microenvironment (see supplemental information).

Finally, to evaluate TOAST in a realistic single-cell-to-spatial integration setting, in a well-structured tissue, under the same strong assumption of regional association of the gradient of expression to the spatial location, we use the results of the alignment of dissociated and spatially resolved data to perform gene expression imputation. Specifically, we integrated an scRNA-seq dataset of 3,005 mouse somatosensory cortex cells from Zeisel et al.[37] with an osmFISH dataset consisting of 4,462

spatially resolved cells and 33 measured genes from Codeluppi et al.[38] We compared TOAST with DeST-OT, Tangram, and gimVI,[39] a probabilistic model specifically designed for the task of imputing gene expression measurements by integrating scRNA-seq and ST. We performed five independent random gene splits, where in each split 80% of the genes shared between the two datasets were selected as observed genes and used for alignment and integration, while the remaining 20% were held out for evaluation. Prediction accuracy was quantified using the Spearman correlation between predicted and true expression values across spatial cells, and results are reported as the mean and standard deviation across the five runs. For OT-based methods, including TOAST and DeST-OT, the predicted spatial expression of a gene was obtained by weighting the single-cell expression profiles according to the learned transport map, yielding a continuous estimate of gene expression at each spatial location. As shown in Figure S6C, TOAST achieves the highest performance on this task.

DISCUSSION

Traditional OT-based alignment methods applied to omics data focus primarily on minimizing discrepancies between molecular profiles while neglecting the critical role of the spatial organization of tissues. The spatial arrangement and functional state of cells are not independent, but they are shaped by interactions with neighboring cells, extracellular matrix components, and local signaling interactions. Intra- and intercellular relationships give rise to spatial patterns that are characteristic of both physiological and pathological conditions. In this study, we introduced a scalable, topography aware FGW OT framework, TOAST, which explicitly incorporates spatial constraints into the classical FGW formulation. By integrating spatial coherence and neighborhood consistency in its objective function, our approach disentangles cell-intrinsic variability from the expression profiles in each cell's neighborhood and effectively models molecular heterogeneity and tissue architecture. Extensive evaluation shows that TOAST leads to biologically meaningful mappings in spatial omics datasets.

On simulated data, our results demonstrate that the inclusion of spatial coherence and neighborhood consistency improves the alignment of both clustered and disorganized tissue regions, with traditional OT methods struggling to capture these nuanced spatial patterns, often leading to biologically implausible mappings. When applied to real ST datasets, TOAST consistently outperforms competing methods across multiple evaluation criteria. In the human DLPFC dataset, our approach achieves the highest accuracy in aligning cortical slices, preserving the integrity of spatially distinct neuronal layers more effectively than traditional OT frameworks. Similarly, in the axolotl brain dataset, TOAST successfully recovers developmental trajectories by accurately aligning cell states across consecutive time points and by modeling the local tissue microenvironment during the alignment process. Beyond ST, we demonstrate the versatility of TOAST by applying it to IMC data from multiple cancer types and by integrating ST with spatial proteomics in a renal carcinoma sample. Our results on the mouse atlas dataset showcase the ability of TOAST to accurately reconstruct spatial organization across anatomical regions. By leveraging spatial constraints, TOAST effectively captures the underlying spatial structure of the mouse embryos, preserving tissue-specific arrangements and improving the biological relevance of cell-cell mappings. Finally, the spatial reconstruction experiments demonstrate that spatial coherence and neighborhood consistency serve as biologically motivated regularizers, biasing the transport solution toward locally smooth and context-preserving alignments, with this effect empirically supported by TOAST's superior performance across downstream integration benchmarks. Beyond the aforementioned applications, our framework provides a principled way to align and integrate multi-omics spatial datasets and could have further implications for understanding developmental trajectories, disease progression, and tissue regeneration.

Limitations of the study

While our framework offers significant improvements over traditional OT-based methods, it is not without limitations. First, TOAST relies on a "balanced" OT formulation, where the total mass transported from one spatial slice to another is constrained to be equal. While suitable for controlled datasets with stable cell type proportions, this assumption may not hold in biological systems where cell proliferation, death, or migration alter mass distributions. In such scenarios, an "unbalanced" OT formulation, which relaxes mass conservation constraints by incorporating additional regularization terms, could provide more flexible and meaningful mappings. Second, our method models pairwise alignment between spatial domains, which may limit its ability to capture more complex temporal trajectories or multi-sample correspondences. A possible extension would be to adopt a multimarginal OT framework, allowing simultaneous alignment of multiple spatial slices or time points. This could enable a more comprehensive modeling of dynamic processes such as development, regeneration, or disease progression.

Looking forward, TOAST opens avenues for a range of downstream tasks beyond alignment of spatially resolved omics, for instance, for reconstruction of spatial trajectories, annotation of disorganized tissue regions, or inferring changes in local tissue microenvironments across conditions or perturbations. In addition, future work could explore adaptive spatial regularization, where neighborhood constraints are dynamically tuned based on local spatial resolution or cell density, enhancing robustness across structurally diverse tissue architectures.

RESOURCE AVAILABILITY

Lead contact

Requests for further information and resources should be directed to and will be fulfilled by the lead contact, Jovan Tanevski (jovan.tanevski@uni-heidelberg.de).

Materials availability

- This study did not generate new biological materials.

Data and code availability

This study used publicly available data, and the original data can be obtained at the following links:

- 10× Visium human dorsolateral prefrontal cortex from https://research.libd.org/spatialLIBD/.

- Axolotl brain Stereo-seq from https://ftp.cngb.org/pub/SciRAID/stomics/STDS0000056/stomics/.
- seqFISH data on mouse organogenesis from https://crukci.shinyapps.io/SpatialMouseAtlas/.
- Imaging mass cytometry data from the Integrated Immunoprofiling of Large Adaptive Cancer patient cohorts (IMMUcan) was accessed through the Bioconductor package *imcdatasets*.
- The source code is accessible at https://github.com/cecca46/TOAST or https://doi.org/10.5281/zenodo.18755751. TOAST is implemented in Python and can be seamlessly integrated into pipelines using Scanpy[40] and Squidpy,[41] facilitating downstream analysis of spatial omics data.
- Any additional information required to reanalyze the data reported in this work paper is available from the lead contact upon request.

ACKNOWLEDGMENTS

We gratefully acknowledge the contribution of Simon Gutwien for his assistance in the preparation of the figures presented in this work. For the purpose of open access, the author has applied a Creative Commons Attribution (CC-BY) license to any author-accepted manuscript version arising from this submission. J.T. is supported by the Bruno und Helene Jöster Stiftung. F.C. acknowledges support from the SynTech programme grant (EP/S024220/1).

AUTHOR CONTRIBUTIONS

Conceptualization, J.T. and F.C.; methodology, F.C., S.B.H., and J.T.; writing – original draft, F.C. and J.T.; writing – review & editing, J.S.-R., P.L., and S.B.H.; supervision, J.T, P.L., and S.B.H.

DECLARATION OF INTERESTS

J.S.-R. reports, in the last 3 years, funding from GSK and Pfizer and fees/honoraria from Travere Therapeutics, Stadapharm, Astex, Pfizer, Grunenthal, Tempus, Moderna, and Owkin.

STAR★METHODS

Detailed methods are provided in the online version of this paper and include the following:

- KEY RESOURCES TABLE
- EXPERIMENTAL MODEL AND STUDY PARTICIPANT DETAILS
- METHOD DETAILS
  - Topography aware fused Gromov-Wasserstein
  - Distance functions
  - One-dimensional simulation
  - Two-dimensional simulation
  - Data preprocessing
  - Related approaches
- QUANTIFICATION AND STATISTICAL ANALYSIS
  - Evaluation metrics
  - Spatial coherence and neighborhood consistency
  - Neighborhood enrichment
  - Statistical testing

SUPPLEMENTAL INFORMATION

Supplemental information can be found online at https://doi.org/10.1016/j.crmeth.2026.101373.

Received: June 29, 2025
Revised: December 22, 2025
Accepted: February 27, 2026
Published: March 30, 2026

REFERENCES

1. Rood, J.E., Maartens, A., Hupalowska, A., Teichmann, S.A., and Regev, A. (2022). Impact of the Human Cell Atlas on medicine. Nat. Med. *28*, 2486–2496.
2. Maynard, K.R., Collado-Torres, L., Weber, L.M., Uytingco, C., Barry, B.K., Williams, S.R., Catallini, J.L., Tran, M.N., Besich, Z., Tippani, M., et al. (2021). Transcriptome-scale spatial gene expression in the human dorsolateral prefrontal cortex. Nat. Neurosci. *24*, 425–436.
3. Beechem, J.M. (2020). High-plex spatially resolved RNA and protein detection using digital spatial profiling: a technology designed for immuno-oncology biomarker discovery and translational research. In Biomarkers for Immunotherapy of Cancer (Springer), pp. 563–583.
4. Eng, C.-H.L., Lawson, M., Zhu, Q., Dries, R., Koulena, N., Takei, Y., Yun, J., Cronin, C., Karp, C., Yuan, G.-C., and Cai, L. (2019). Transcriptome-scale super-resolved imaging in tissues by RNA seqFISH+. Nature *568*, 235–239.
5. Chen, K.H., Boettiger, A.N., Moffitt, J.R., Wang, S., and Zhuang, X. (2015). Spatially resolved, highly multiplexed RNA profiling in single cells. Science *348*, aaa6090.
6. Rodriques, S.G., Stickels, R.R., Goeva, A., Martin, C.A., Murray, E., Vanderburg, C.R., Welch, J., Chen, L.M., Chen, F., and Macosko, E.Z. (2019). Slide-seq: A scalable technology for measuring genome-wide expression at high spatial resolution. Science *363*, 1463–1467.
7. Palla, G., Fischer, D.S., Regev, A., and Theis, F.J. (2022). Spatial components of molecular tissue biology. Nat. Biotechnol. *40*, 308–318.
8. Schiebinger, G., Shu, J., Tabaka, M., Cleary, B., Subramanian, V., Solomon, A., Gould, J., Liu, S., Lin, S., Berube, P., et al. (2019). Optimal-transport analysis of single-cell gene expression identifies developmental trajectories in reprogramming. Cell *176*, 1517–1943.
9. Galichon, A. (2018). Optimal Transport Methods in Economics (Princeton University Press).
10. Solomon, J., De Goes, F., Peyré, G., Cuturi, M., Butscher, A., Nguyen, A., Du, T., and Guibas, L. (2015). Convolutional wasserstein distances: Efficient optimal transportation on geometric domains. ACM Trans. Graph. *34*, 1–11.
11. Courty, N., Flamary, R., Tuia, D., and Rakotomamonjy, A. (2017). Optimal transport for domain adaptation. IEEE Trans. Pattern Anal. Mach. Intell. *39*, 1853–1865.
12. Bunne, C., Schiebinger, G., Krause, A., Regev, A., and Cuturi, M. (2024). Optimal transport for single-cell and spatial omics. Nat. Rev. Methods Primers *4*, 58. https://doi.org/10.1038/s43586-024-00334-2.
13. Villani, C. (2021). Topics in Optimal Transportation (American Mathematical Soc.).
14. Peyré, G., and Cuturi, M. (2019). Computational optimal transport: With applications to data science. Foundations and Trends® in Machine Learning *11*, 355–607.
15. Zeira, R., Land, M., Strzalkowski, A., and Raphael, B.J. (2022). Alignment and integration of spatial transcriptomics data. Nat. Methods *19*, 567–575. https://doi.org/10.1038/s41592-022-01459-6.
16. Liu, X., Zeira, R., and Raphael, B.J. (2023). Partial alignment of multislice spatially resolved transcriptomics data. Genome Res. *33*, 1124–1132.
17. Halmos, P., Liu, X., Gold, J., Chen, F., Ding, L., and Raphael, B.J. (2025). DeST-OT: Alignment of spatiotemporal transcriptomics data. Cell Syst. *16*, 101160.
18. Rahimi, A., Vale-Silva, L.A., Fälth Savitski, M., Tanevski, J., and Saez-Rodriguez, J. (2024). DOT: a flexible multi-objective optimization framework for transferring features across single-cell and spatial omics. Nat. Commun. *15*, 4994. https://doi.org/10.1038/s41467-024-48868-z.
19. Klein, D., Palla, G., Lange, M., Klein, M., Piran, Z., Gander, M., Meng-Papaxanthos, L., Sterr, M., Saber, L., Jing, C., et al. (2025). Mapping cells through time and space with moscot. Nature *638*, 1065–1075.

20. Schürch, C.M., Bhate, S.S., Barlow, G.L., Phillips, D.J., Noti, L., Zlobec, I., Chu, P., Black, S., Demeter, J., McIlwain, D.R., et al. (2020). Coordinated cellular neighborhoods orchestrate antitumoral immunity at the colorectal cancer invasive front. Cell *182*, 1341–1359.e19.

21. Pelka, K., Hofree, M., Chen, J.H., Sarkizova, S., Pirl, J.D., Jorgji, V., Bejnood, A., Dionne, D., Ge, W.H., Xu, K.H., et al. (2021). Spatially organized multicellular immune hubs in human colorectal cancer. Cell *184*, 4734–4752.e20.

22. Wei, X., Fu, S., Li, H., Liu, Y., Wang, S., Feng, W., Yang, Y., Liu, X., Zeng, Y.-Y., Cheng, M., et al. (2022). Single-cell Stereo-seq reveals induced progenitor cells involved in axolotl brain regeneration. Science *377*, eabp9444.

23. Rao, A., Barkley, D., França, G.S., and Yanai, I. (2021). Exploring tissue architecture using spatial transcriptomics. Nature *596*, 211–220.

24. Tanevski, J., Flores, R.O.R., Gabor, A., Schapiro, D., and Saez-Rodriguez, J. (2022). Explainable multiview framework for dissecting spatial relationships from highly multiplexed data. Genome Biol. *23*, 97.

25. Tanevski, J., Vulliard, L., Ibarra-Arellano, M.A., Schapiro, D., Hartmann, F.J., and Saez-Rodriguez, J. (2025). Learning tissue representation by identification of persistent local patterns in spatial omics data. Nat. Commun. *16*, 4071.

26. Greenwald, A.C., Darnell, N.G., Hoefflin, R., Simkin, D., Mount, C.W., Gonzalez Castro, L.N., Harnik, Y., Dumont, S., Hirsch, D., Nomura, M., et al. (2024). Integrative spatial analysis reveals a multi-layered organization of glioblastoma. Cell *187*, 2485–2501.e26.

27. Cuturi, M. (2013). Sinkhorn Distances: Lightspeed Computation of Optimal Transport. In Advances in Neural Information Processing Systems, C.J. Burges, L. Bottou, M. Welling, Z. Ghahramani, and K.Q. Weinberger, eds. (Curran Associates, Inc.).

28. Biancalani, T., Scalia, G., Buffoni, L., Avasthi, R., Lu, Z., Sanger, A., Tokcan, N., Vanderburg, C.R., Segerstolpe, Å., Zhang, M., et al. (2021). Deep learning and alignment of spatially resolved single-cell transcriptomes with Tangram. Nat. Methods *18*, 1352–1362.

29. Nitzan, M., Karaiskos, N., Friedman, N., and Rajewsky, N. (2019). Gene expression cartography. Nature *576*, 132–137. https://doi.org/10.1038/s41586-019-1773-3.

30. Traag, V.A., Waltman, L., and Van Eck, N.J. (2019). From Louvain to Leiden: guaranteeing well-connected communities. Sci. Rep. *9*, 5233.

31. Eling, N., Dorier, J., Rusakiewicz, S., Liechti, R., Devanand, P., Daniel, M., Windhager, J., Fernandez, B.P., Deglise, S., Despland, L., et al. (2025). Multi-modal image analysis for large scale cancer tissue studies within IMMUcan. Cell Rep. Methods *5*, 101170.

32. 10x Genomics (2025). Xenium In Situ Gene and Protein Expression data for FFPE Human Renal Cell Carcinoma

33. Tanevski, J., Nguyen, T., Truong, B., Karaiskos, N., Ahsen, M.E., Zhang, X., Shu, C., Xu, K., Liang, X., Hu, Y., et al. (2020). Gene selection for optimal prediction of cell position in tissues from single-cell transcriptomics data. Life Sci. Alliance *3*, e202000867.

34. Lohoff, T., Ghazanfar, S., Missarova, A., Koulena, N., Pierson, N., Griffiths, J.A., Bardot, E.S., Eng, C.H.L., Tyser, R.C.V., Argelaguet, R., et al. (2022). Integration of spatial and single-cell transcriptomic data elucidates mouse organogenesis. Nat. Biotechnol. *40*, 74–85.

35. Hao, M., Luo, E., Chen, Y., Wu, Y., Li, C., Chen, S., Gao, H., Bian, H., Gu, J., Wei, L., and Zhang, X. (2024). STEM enables mapping of single-cell and spatial transcriptomics data with transfer learning. Commun. Biol. *7*, 56.

36. Ji, A.L., Rubin, A.J., Thrane, K., Jiang, S., Reynolds, D.L., Meyers, R.M., Guo, M.G., George, B.M., Mollbrink, A., Bergenstråhle, J., et al. (2020). Multimodal analysis of composition and spatial architecture in human squamous cell carcinoma. Cell *182*, 1661–1662.

37. Zeisel, A., Muñoz-Manchado, A.B., Codeluppi, S., Lönnerberg, P., La Manno, G., Juréus, A., Marques, S., Munguba, H., He, L., Betsholtz, C., et al. (2015). Cell types in the mouse cortex and hippocampus revealed by single-cell RNA-seq. Science *347*, 1138–1142.

38. Codeluppi, S., Borm, L.E., Zeisel, A., La Manno, G., van Lunteren, J.A., Svensson, C.I., and Linnarsson, S. (2018). Spatial organization of the somatosensory cortex revealed by osmFISH. Nat. Methods *15*, 932–935.

39. Lopez, R., Nazaret, A., Langevin, M., Samaran, J., Regier, J., Jordan, M.I., and Yosef, N. (2019). A joint model of unpaired data from scRNA-seq and spatial transcriptomics for imputing missing gene expression measurements. arXiv, . Preprint at. https://doi.org/10.48550/arXiv.1905.02269.

40. Wolf, F.A., Angerer, P., and Theis, F.J. (2018). SCANPY: large-scale single-cell gene expression data analysis. Genome Biol. *19*, 15.

41. Palla, G., Spitzer, H., Klein, M., Fischer, D., Schaar, A.C., Kuemmerle, L.B., Rybakov, S., Ibarra, I.L., Holmberg, O., Virshup, I., et al. (2022). Squidpy: a scalable framework for spatial omics analysis. Nat. Methods *19*, 171–178.

42. Flamary, R., Courty, N., Gramfort, A., Alaya, M.Z., Boisbunon, A., Chambon, S., Chapel, L., Corenflos, A., Fatras, K., Fournier, N., et al. (2021). POT: Python Optimal Transport. J. Mach. Learn. Res. *22*, 1–8.

43. Sinkhorn, R., and Knopp, P. (1967). Concerning nonnegative matrices and doubly stochastic matrices. Pac. J. Math. *21*, 343–348.

44. Peyré, G., Cuturi, M., and Solomon, J. (2016). Gromov-wasserstein averaging of kernel and distance matrices. In International conference on machine learning (PMLR), pp. 2664–2672.

45. Benamou, J.-D., Carlier, G., Cuturi, M., Nenna, L., and Peyré, G. (2015). Iterative Bregman projections for regularized transportation problems. SIAM J. Sci. Comput. *37*, A1111–A1138.

STAR★METHODS

KEY RESOURCES TABLE

REAGENT or RESOURCE	SOURCE	IDENTIFIER
Deposited data		
Mouse somatosensory cortex scRNA-seq dataset	Zeisel et al.[37]	GEO: GSE60361
Mouse cortex osmFISH dataset	Codeluppi et al.[38]	http://linnarssonlab.org/osmFISH
Human dorsolateral prefrontal cortex Visium dataset	spatialLIBD	https://research.libd.org/spatialLIBD/
Axolotl brain Stereo-seq dataset	Wei et al.[22]	https://ftp.cngb.org/pub/SciRAID/stomics/STDS0000056/stomics/
Mouse embryo seqFISH dataset	Lohoff et al.[34]	https://crukci.shinyapps.io/SpatialMouseAtlas/
Imaging Mass Cytometry dataset	IMMUcan consortium	https://www.bioconductor.org/packages/release/data/experiment/html/imcdatasets.html
Xenium transcript–protein dataset	10x Genomics	https://www.10xgenomics.com
Software and algorithms		
TOAST	This paper	https://github.com/cecca46/TOAST
DeST-OT	Halmos et al.[17]	https://github.com/raphael-group/DeST_OT
Tangram	Biancalani et al.[28]	https://github.com/broadinstitute/Tangram
gimVI	Lopez et al.[39]	https://github.com/scverse/scvi-tools
POT (Python Optimal Transport)	Flamary et al.[42]	https://pythonot.github.io

EXPERIMENTAL MODEL AND STUDY PARTICIPANT DETAILS

This study did not involve experimental models or human subjects.

METHOD DETAILS

Topography aware fused Gromov-Wasserstein

The Fused Gromov-Wasserstein (FGW) distance is an optimal transport framework that aligns distributions based on both *feature similarity* and *structural similarity*. The usual formulation of FGW is

$$\Gamma(\boldsymbol{\Pi}) = (1-\alpha)\langle \boldsymbol{\Pi}, \mathbf{M}\rangle_F + \alpha \sum_{i,j,k,l} L\left(C_{1,i,k}, C_{2,j,l}\right)\boldsymbol{\Pi}_{i,j}\boldsymbol{\Pi}_{k,l} - \varepsilon H(\boldsymbol{\Pi}) \quad \text{(Equation 7)}$$

where $H(\boldsymbol{\Pi}) = \sum_{i,j}\boldsymbol{\Pi}_{i,j}\log\left(\boldsymbol{\Pi}_{i,j}\right)$ is the entropy of the transport matrix and $\varepsilon > 0$ controls the strength of this entropic regularization. In this work, we extend FGW to

$$\Gamma(\boldsymbol{\Pi}) = (1-\alpha)\langle \Pi, \mathbf{M}\rangle_F + \frac{\alpha}{3}\left[\sum_{i,j,k,l} L\left(C_{1,i,k}, C_{2,j,l}\right)\boldsymbol{\Pi}_{i,j}\boldsymbol{\Pi}_{k,l} + \langle \boldsymbol{\Pi}, \mathbf{C_3}\rangle_F + \langle \boldsymbol{\Pi}, \mathbf{C_4}\rangle_F\right] - \epsilon H(\boldsymbol{\Pi}) \quad \text{(Equation 8)}$$

Given some cost matrix $\mathbf{M}$, where $\mathbf{M}_{ij}$ represents the transportation cost between positions indexed by i and j optimal transport seeks to find a coupling between two probability distributions. Let $p \in \Delta^n$ and $q \in \Delta^m$ be discrete probability distributions over the source and target spaces, respectively, where p_i and q_j denote the probability mass associated with locations indexed by i and j. Here, Δ^n represents the n-dimensional probability simplex defined as

$$\Delta^n = \left\{ p \in R_+^n \,\middle|\, \sum_{i=1}^{n} p_i = 1 \right\}. \quad \text{(Equation 9)}$$

This ensures that the entries of p and q are non-negative and sum to one, making them valid probability distributions. Then, the solution of the entropically normalized optimal transport between p and q is the convex optimization problem

$$\boldsymbol{\Pi}^*(\boldsymbol{M},p,q) = \arg\min_{\Pi \in \mathcal{C}_{p,q}} \langle \boldsymbol{\Pi}, \boldsymbol{M} \rangle_F - \epsilon H(\boldsymbol{\Pi}), \quad \text{(Equation 10)}$$

where $\mathcal{C}_{p,q} = \{\boldsymbol{\Pi} \in (R_+)^{n \times m}; \boldsymbol{\Pi} 1_{n_2} = \mathbf{p}, \boldsymbol{\Pi}^\top 1_{n_1} = \mathbf{q}\}$ is the set of possible couplings between p and q. As shown in Cuturi,[27] the solution is $\Pi^*(M,p,q) = \text{diag}(a) K \text{diag}(b)$ where $K = e^{-\frac{M}{\epsilon}} \in R^{n \times m}$ is the Gibbs kernel associated to $\boldsymbol{M}$ and (a,b) are computed as

$$a \leftarrow \frac{p}{Kb} \quad \text{and} \quad b \leftarrow \frac{q}{K^T a} \quad \text{(Equation 11)}$$

using Sinkhorn iterations.[43] The Gromov-Wasserstein (GW) term measures the discrepancy between the two distance matrices $C_1 \in R^{n \times n}$ and $C_2 \in R^{m \times m}$ defined on space p and q, respectively. For simplicity define

$$\mathcal{E}_{\mathcal{C}_1,\mathcal{C}_2}(\boldsymbol{\Pi}) = \sum_{i,j,k,l} L(C_{1,i,k}, C_{2,j,l}) \boldsymbol{\Pi}_{i,j} \boldsymbol{\Pi}_{k,l}, \quad \text{(Equation 12)}$$

where L is some loss function to account for the discrepancy between the distance matrices. Using the 4-way tensor

$$\mathcal{L}(\boldsymbol{C_1}, \boldsymbol{C_2}) = \left(L(C_{1,i,k}, C_{2,j,l})\right)_{i,j,k,l}, \quad \text{(Equation 13)}$$

we have $\mathcal{E}_{\mathcal{C}_1,\mathcal{C}_2}(\boldsymbol{\Pi}) = \langle \mathcal{L}(\boldsymbol{C_1}, \boldsymbol{C_2}) \otimes \boldsymbol{\Pi}, \boldsymbol{\Pi} \rangle$, where $\otimes$ defines tensor-matrix multiplication. In Peyré et al.,[44] the authors showed how to efficiently compute $\mathcal{L}(\boldsymbol{C_1}, \boldsymbol{C_2}) \otimes \Pi$ for a general class of loss functions. We define the entropic approximation of the Gromov-Wasserstein formulation in Equation 8 as

$$GW(\boldsymbol{C_1}, \boldsymbol{C_2}, p, q) = \min_{\Pi \in \mathcal{C}_{p,q}} \mathcal{E}_{\mathcal{C}_1,\mathcal{C}_2}(\boldsymbol{\Pi}) - \epsilon H(\boldsymbol{\Pi}). \quad \text{(Equation 14)}$$

Equation 14 is a non-convex optimization problem that can be solved using projected gradient descent (PGD), where the projections are computed according to the Kullback-Leibler (KL) metric. An iteration of this algorithm reads as

$$\boldsymbol{\Pi} \leftarrow Proj^{KL}_{\mathcal{C}_{p,q}}\left(\boldsymbol{\Pi} \odot e^{-\tau\left(\nabla \mathcal{E}_{\boldsymbol{C_1},\boldsymbol{C_2}}(\boldsymbol{\Pi}) - \epsilon \nabla H(\boldsymbol{\Pi})\right)}\right), \quad \text{(Equation 15)}$$

where τ is the step size, and the KL projection of any matrix K is given by:

$$\text{Proj}^{\text{KL}}_{\mathcal{C}_{p,q}}(K) = \arg\min_{\boldsymbol{\Pi}' \in \mathcal{C}_{p,q}} \text{KL}(\boldsymbol{\Pi}'|K). \quad \text{(Equation 16)}$$

Essentially, Equation 16 projects the transport plan onto the feasible set using KL divergence, ensuring valid marginals while preserving entropy regularization. As shown in Peyré et al., Benamou et al.,[44,45] for small enough steps of τ, the projection is just the solution to the regularized transport problem in Equation 10. Hence, minimizing the problem in Equation 8, reduces to the following Sinkhorn iterations:

$$(\widehat{\boldsymbol{\Pi}})^{t+1} \leftarrow e\left(-\frac{\nabla_{\boldsymbol{\Pi}}\Gamma}{\varepsilon}\right) \odot \boldsymbol{\Pi}^t \quad \text{where} \quad \nabla_{\boldsymbol{\Pi}}\Gamma = (1-\alpha)\boldsymbol{M} + \frac{\alpha}{3}\left(\mathcal{L}(\boldsymbol{C_1}, \boldsymbol{C_2}) \otimes \boldsymbol{\Pi} + \boldsymbol{C_3} + \boldsymbol{C_4}\right) \quad \text{(Equation 17)}$$

$$a^{(t+1)} \leftarrow \frac{p}{\widehat{\boldsymbol{\Pi}}\, b^{(t)}} \quad \text{and} \quad b^{(t+1)} \leftarrow \frac{q}{(\widehat{\boldsymbol{\Pi}})^T a^{(t+1)}} \quad \text{(Equation 18)}$$

The transport plan is iteratively computed from Equations 17 and 18 as

$$\boldsymbol{\Pi}^{t+1} = \text{diag}(a)\, (\widehat{\boldsymbol{\Pi}})^{t+1}\, \text{diag}(b)$$

Distance functions

For our topography aware Fused-Gromov Wasserstein formulation in Equation 5, we define multiple loss functions, operating in the principal component space Z^p, to compute distances between cells in the source and target domains. In detail, the cost matrix $\boldsymbol{M}$ is computed as

$$M_{i,j} = |\boldsymbol{z_i} - \boldsymbol{z_j}|_{\boldsymbol{2}},$$

where $\boldsymbol{z_i}$ and $\boldsymbol{z_j}$ are the PCA embeddings for a point in the source and target domain, respectively. The matrix C_3, enforcing spatial coherence, is computed as

$$C_{3,i,j} = |z_i - z_j|.$$

Similarly, C_4, designed to model neighborhood consistency, is given by:

$$C_{4,i,j} = |z_i - z_j|_2.$$

The matrices $C_{1,i,k}$ and $C_{2,j,l}$, which define structural relationships between points in the source and target domains, are computed as

$$C_{1,i,k} = |z_i - z_k|_2, C_{2,j,l} = |z_j - z_l|_2.$$

Finally, the function L, capturing pairwise dependencies between transported distributions, is defined as

$$L(C_{1,i,k}, C_{2,j,l}) = (C_{1,i,k} - C_{2,j,l})^2.$$

One-dimensional simulation

To simulate the one-dimensional spatial transcriptomics data, we followed the approach described in Halmost et al.[17] We considered a pair of one-dimensional tissue slices, each consisting of 101 spatially ordered spots, spanning from -N to N, where N = 50. The spatial coordinates for both slices are represented by:

$$S^{(1)} = S^{(2)} = \{-N, (N-1), \ldots, -1, 0, 1, \ldots, (N-1), N\}^{\mathrm{T}}.$$

The spots are organized in clustered or disorganized regions: clustered regions only contain cell type A or cell type B, while disorganized regions exhibit alternation of the two cell types. The partition for slice 1 is given by:

$$P_1(A) = \{-N, \ldots, w_1\},$$

$$P_1(AB) = \{w_1 + 1, \ldots, w_2\},$$

$$P_1(B) = \{w_2 + 1, \ldots, N\},$$

where $w_1 = -20$ and $w_2 = 20$ mark the boundaries of the clustered and disorganized regions. Similarly, the partition for slice 2 is:

$$P_2(A) = \{-N, \ldots, w_3\},$$

$$P_2(AB) = \{w_3 + 1, \ldots, w_4\},$$

$$P_2(B) = \{w_4 + 1, \ldots, N\}$$

where $w_3 = -15$ and $w_4 = 10$ define the boundaries in the second slice.

Random vectors v_1,v_2,v_3,v_4, independently and uniformly sampled from the unit sphere in $\mathbb{R}^4$, are used to create orthogonal feature gradients for cell types A and B. The features are constructed as

$$\tilde{v_1} = v_1 \circ 0,$$

$$\tilde{v_2} = v_2 \circ 0,$$

$$\tilde{v_3} = 0 \circ v_3,$$

$$\tilde{v_4} = 0 \circ v_4$$

where $0 \in \mathbb{R}^4$ is the zero vector, and $\circ$ denotes vector concatenation. Features are then generated by linearly interpolating between two fixed vectors:

For cell type A: $\tilde{v}_1$ at $-N$ and $\tilde{v}_2$ *at* w_1(or w_3)

For cell type B: $\tilde{v}_3$ at w_2 (or w_4) and $\tilde{v}_4$ *at N*

After generating the features, Gaussian noise is added to simulate variability. The noise is sampled from a normal distribution with mean $\mu = 0$ and standard deviation $\sigma = 0.1$. This setup ensures that features for cell types A and B remained orthogonal, with each region exhibiting distinct gradients along four-dimensional feature spaces, with added noise reflecting natural variability.

Two-dimensional simulation

To extend the one-dimensional model to two-dimensions, we define a centered, circular ellipse E of radius r = 20, represented as

$$E = \left\{ s \equiv (x, y) \in \mathbb{R}^2 : \frac{x^2}{r^2} + \frac{y^2}{r^2} \le 1 \right\}.$$

Next, we define T, a subset of the integer square lattice $\mathbb{Z}^2$, consisting of all integers pairs whose sum is odd:

$$T = \{(x, y) \in \mathbb{Z}^2 : (x + y) \bmod 2 = 1\}.$$

This results in a triangular lattice structure, which mimics the spatial organization of spatial transcriptomics data produced by platforms like 10X Genomics Visium.[2] The spatial coordinates for both tissue slices, denoted $S^{(1)} = S^{(2)} = S$, are given by the intersection $E \cap T$, representing spots within the circular ellipse. To partition each slice into clustered and disorganized regions, we define pivot lines for the y-coordinates. For slice 1, the pivot lines are $y_1^1 = -5$ and $y_2^1 = 2$. For slice 2, the pivot lines are $y_1^2 = 0$ and $y_2^2 = 7$. Based on these pivots, we define three areas for slice 1:

$$P_1(A) = \left\{s = (x,y) \in T : y < y_1^1\right\}$$

$$P_1(AB) = \left\{s = (x,y) \in T : y_1^1 \le y \le y_2^1\right\}$$

$$P_1(B) = \left\{s = (x,y) \in T : y > y_2^1\right\}$$

For slice 2, the partitions follow a similar structure:

$$P_2(A) = \left\{s = (x,y) \in T : y < y_1^2\right\}$$

$$P_2(AB) = \left\{s = (x,y) \in T : y_1^2 \le y \le y_2^2\right\}$$

$$P_2(B) = \left\{s = (x,y) \in T : y > y_2^2\right\}$$

For each slice i, let $R_i(A)$, $R_i(B)$ and $R_i(AB)$ denote the smallest bounding rectangles for the clustered and disorganized regions $P_i(A)$, $P_i(B)$ and $P_i(AB)$ respectively. For feature assignment, we use the first four unit vectors in the standard basis of $\mathbb{R}^8$, denoted e_1,e_2,e_3,e_4, assigned to cell type A, and the last four vectors, e_5,e_6,e_7,e_8, assigned to cell type B. At each spot $s=(x,y)$ within a bounding rectangle $R = [x_{min}, x_{max}] \times [y_{min}, y_{max}]$, the feature vector is determined as a convex combination of the features decorating the top and bottom sides of R, plus the features decorating the left and right sides of R. The coefficients λ_x and λ_y are defined as

$$\lambda_x = \frac{x - x_{min}}{x_{max} - x_{min}}, \lambda_y = \frac{y - y_{min}}{y_{max} - y_{min}}.$$

For cell type A, the feature vector is:

$$f_A(x,y) = \lambda_x f_{x,L} + (1 - \lambda_x) f_{x,R} + \lambda_y f_{y,T} + \left(1 - \lambda_y\right) f_{y,B}.$$

For cell type B, the feature vector is:

$$g_B(x,y) = \lambda_x g_{x,L} + (1 - \lambda_x) g_{x,R} + \lambda_y g_{y,T} + \left(1 - \lambda_y\right) g_{y,B}.$$

To simulate variability, Gaussian noise, sampled from a normal distribution with mean $\mu = 0$ and standard deviation $\sigma = 0.1$, was added to the features.

Data preprocessing

We preprocessed the data using the Scanpy Python package.[40] Specifically, we performed library size normalization (sc.pp.normalize_total) followed by log-transformation (sc.pp.log1p). Principal component analysis (PCA) was then applied to reduce dimensionality.

Related approaches

DeST-OT: Developmental spatiotemporal Optimal Transport (DeST-OT)[17] is a semi-relaxed optimal transport method introduced to model cellular growth, death, and differentiation processes. DeST-OT generates mapping probabilities for each cell in one slice to each cell in the other slice. Therefore, we selected the positions with the maximum probability as the prediction in our comparison experiments. We evaluated DeST-OT using the default parameters suggested by the authors but setting the optimization problem as a balanced one to fairly compare to the other methods.

NovoSpaRc: NovoSpaRc[29] is an optimal transport method which maps cells to tissue locations. The predefined locations for the mapping were constructed based on the reference ST dataset. Following the NovoSpaRc pipeline, we first identified marker genes from the reference dataset and then performed the mapping process based on these markers. We run NovoSpaRc using the default parameters suggested by the authors.

Tangram: Tangram[28] is a deep learning framework that aligns single-cell and single-nucleus RNA-seq data to various forms of spatial data collected from the same region. Similarly to the other methods, Tangram generates mapping probabilities for each cell. We run Tangram using the default parameters suggested by the authors for 200 epochs.

Paste2: Paste[15] was originally introduced as a balanced optimal transport method to align and integrate ST data from multiple adjacent tissue slices. Paste2[16] was introduced as an extension to Paste as a method for partial alignment of slices. Paste2 is based

on the Fused Gromov-Wasserstein Optimal Transport problem. We run Paste2 with the default parameters suggested by the authors but setting the slice overlap argument to 1 (all mass is transported from the source slice to the target slice).

FGW: The traditional Fused Gromov-Wasserstein formulation was implemented in Python using the POT package.[11]

QUANTIFICATION AND STATISTICAL ANALYSIS

Evaluation metrics

For all comparisons to the related approaches, we computed an accuracy score based on the maximum probability assignment for each cell. In detail, for each cell i in the source domain, we identified its most probable match in the target domain given a pairwise alignment matrix $\Pi = [\pi_{i,j}]$ generated by a method as

$$j^* = arg\,max_j\, \pi_{i,j}.$$

Using this assignment, we defined the accuracy as the proportion of correctly matched cells, where correctness was evaluated based on known biological annotations (for example, cell types). To explore the ability of the models to reconstruct the local spatial heterogeneity, we also quantified the discrepancy of cell type distributions between source and aligned slices using the Jensen-Shannon Divergence (JSD). Given two probability distributions P and Q, JSD is defined as

$$\mathrm{JSD}(P,Q) = \frac{1}{2}D_{\mathrm{KL}}(P|M) + \frac{1}{2}D_{\mathrm{KL}}(Q|M),$$

where $M = \frac{1}{2}(P+Q)$ is the average distribution, and $D_{\mathrm{KL}}(P|Q)$ denotes the Kullback-Leibler (KL) divergence:

$$D_{\mathrm{KL}}(P|Q) = \sum_i P_i \log\frac{P_i}{Q_i}.$$

For each pair of aligned cells, we quantified the JSD of their nearest 20 neighbors, capturing local discrepancies in cell type distributions. The overall JSD score of a method was then computed as the mean JSD across all aligned cells:

$$\mathrm{JSD}_{\mathrm{method}} = \frac{1}{N}\sum_{i=1}^{N}\mathrm{JSD}(P_i,Q_i),$$

where N is the total number of aligneds, and $\mathrm{JSD}(P_i,Q_i)$ represents the JSD between the local cell type distributions of the i-th aligned cell in the source and target domains. A lower overall JSD score indicates that the method preserves the local spatial organization of cell types more effectively. For the spatial reconstruction experiments on the Spatial Mouse Atlas, given the continuous nature of the predictions (spatial locations), we compared the models using Mean Absolute Error (MAE):

$$\mathrm{MAE} = \frac{1}{N}\sum_{i=1}^{N}\left|\hat{x}_i - x_i\right|_1,$$

where $\hat{x}_i$ and x_i represent the predicted and ground truth spatial coordinates of cell i, respectively, and $|\cdot|_1$

denotes the L_1-norm. Additionally, we computed the Pearson correlation coefficient to assess the linear relationship between predicted and true spatial locations:

$$r = \frac{\sum_{i=1}^{N}(\hat{x}_i - \bar{\hat{x}})(x_i - \bar{x})}{\sqrt{\sum_{i=1}^{N}(\hat{x}_i - \bar{\hat{x}})^2}\sqrt{\sum_{i=1}^{N}(x_i - \bar{x})^2}},$$

where $\bar{\hat{x}}$ and $\bar{x}$ are the means of the predicted and ground truth coordinates, respectively.

Spatial coherence and neighborhood consistency

To compute the spatial coherence and neighborhood consistency matrices in Equation 5, we build a k-nearest neighbor graph from the (x,y) coordinates of each slice. Therefore, for each cell, the spatial coherence and neighborhood consistency values are simply defined as in Equation 2 and Equation 3, respectively. We set the value of k to 10 for the Spatial Mouse Atlas and Human Dorsolateral Prefron Cortex dataset, and 5 for the Axolotl Brain Stereo-seq dataset. On the latter, given the lack of spatial coordinated for the single-cell (SC) data, we computed the spatial coherence scores and neighborhood averages by constructing a k-nearest neighbors graph based on the SC expression distances rather than spatial coordinates. In all real-data analyses, unless otherwise stated, we selected $\alpha = 0.5$, providing a balanced weighting between molecular similarity and spatial topology. While this choice provided

stable and interpretable results across datasets, we acknowledge that α can be treated as a hyperparameter to be tuned using a small validation slice or selected based on the desired emphasis: lower α favors molecular similarity, while higher α emphasizes structural preservation.

Neighborhood enrichment

We performed neighborhood enrichment on the cell types from the Spatial Mouse Atlas using nhood_enrichment from the Squidpy Python package.[41] This function computes a neighborhood enrichment *Z* score for each cell type by a permutation test. We used the function's default parameters.

Statistical testing

Pairwise method comparisons were performed using Wilcoxon rank-sum tests where applicable. No data points were excluded from the analysis.

Topography Aware Optimal Transport for Alignment of Spatial Omics Data.

Cell Reports Methods, Volume *6*

Supplemental information

Topography-aware optimal transport for alignment of spatial omics data

Francesco Ceccarelli, Pietro Liò, Julio Saez-Rodriguez, Sean B. Holden, and Jovan Tanevski

SUPPLEMENTAL INFORMATION
Topography Aware Optimal Transport for Alignment of Spatial Omics Data

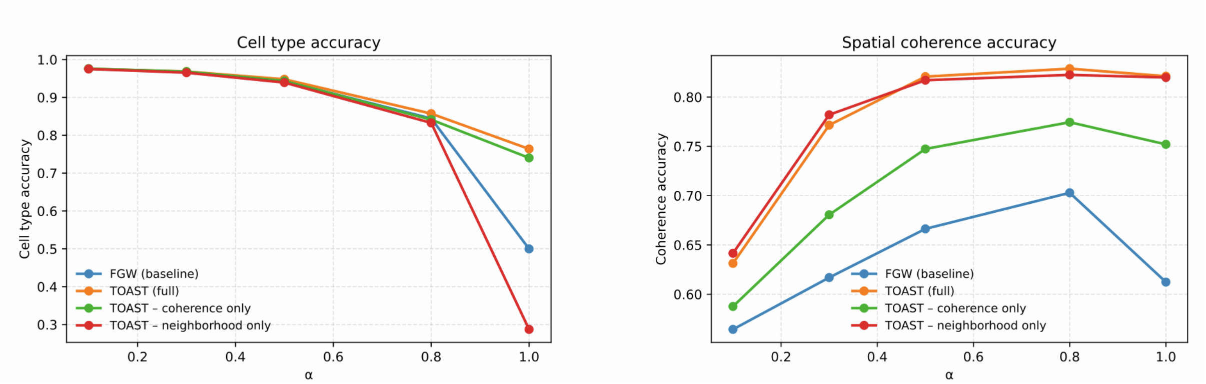

Figure S1: Spatial terms ablation study, related to **Figure 2**. Comparison between standard FGW, TOAST with both spatial coherence and neighborhood consistency, TOAST with only spatial coherence and TOAST with only neighborhood consistency.

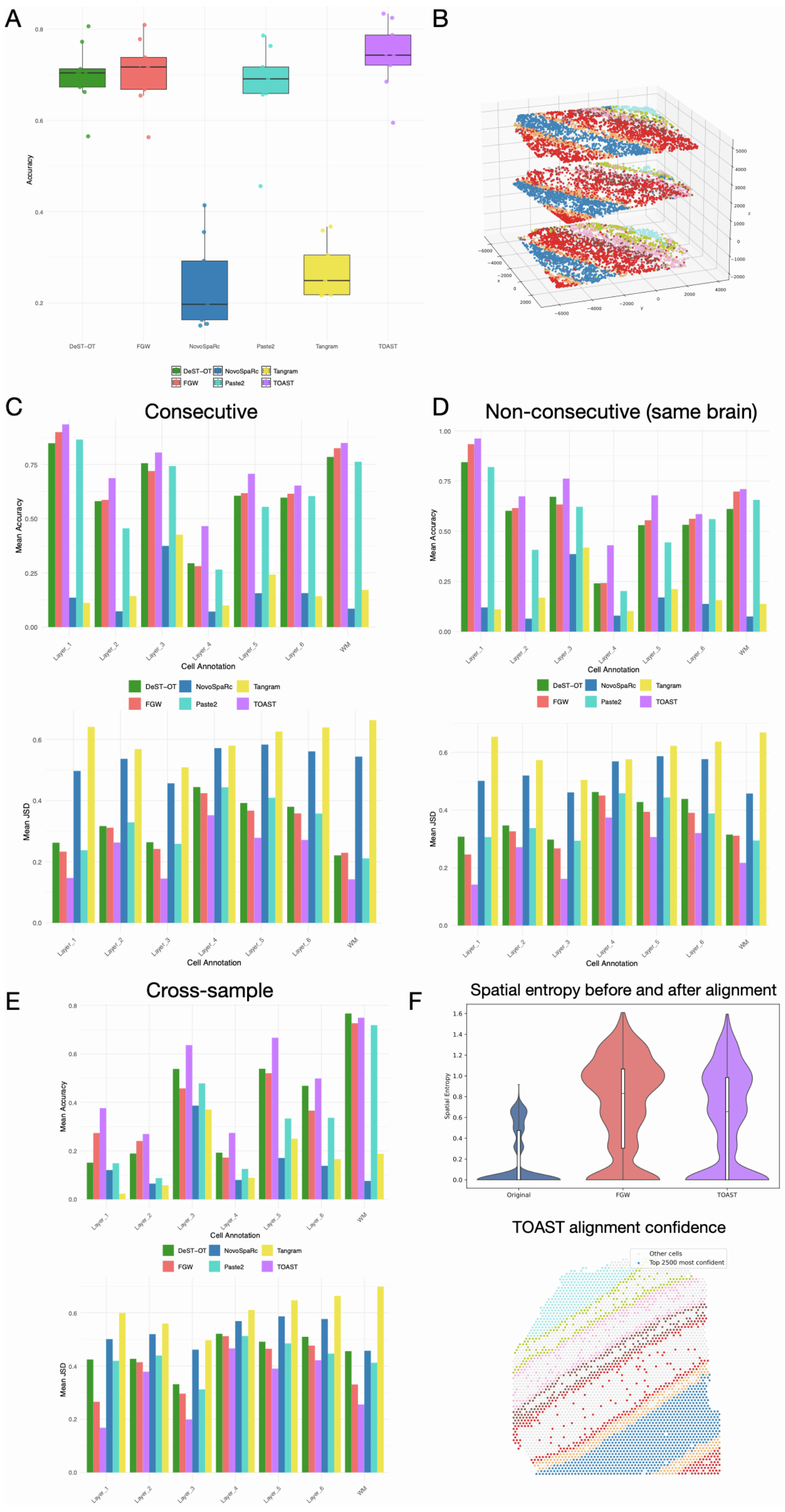

Figure S2: TOAST alignment on DLPFC data, related to **Figure 3**. **A.)** Quantitative comparison of pairwise alignment across consecutive slices for FGW, DeST-OT, Tangram, Paste2, NovoSparc and TOAST with cell labels obtained from unsupervised clustering. **B.)** Stacked 3D alignment of tissue slices of a DLPFC sample after alignment with TOAST. **C.)** Quantitative comparison of pairwise alignment across all consecutive, **D.)** non-consecutive, and **E.)** cross-sample slices in terms of accuracy and Jensen-Shannon divergence. **F.) Upper panel**: Violin plots showing the distribution of spatial entropy for the source slice prior to alignment and after reconstruction using FGW and TOAST. TOAST preserves the original spatial organization more faithfully than FGW. **Lower panel**: TOAST alignment confidence for a pairwise alignment. We display the top 2,500 most confidently aligned cells (colored by ground-truth annotation), while lower-confidence cells are shown in light gray. Distinct cell populations exhibit different confidence profiles. These maps guide downstream analyses by indicating which aligned cells are reliable, and which warrant further biological inspection.

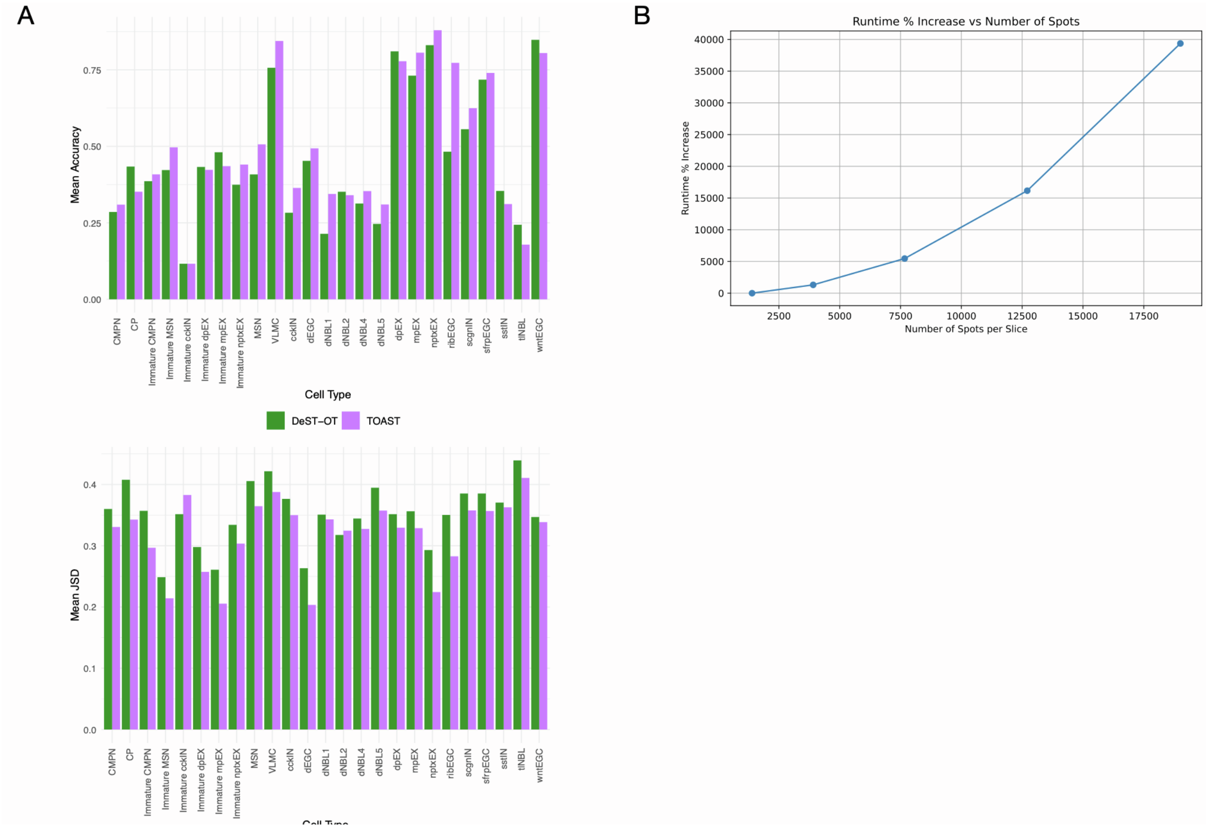

Figure S3: Cell type performance and runtime analysis, related to **Figure 4 and Figure 5**. **A.)** Quantitative comparison of pairwise alignment on Stereo-seq data across all consecutive slices for TOAST and DeST-OT evaluated in terms of accuracy and Jensen-Shannon divergence for each annotated cell type. **B.)** Percentage increase in runtime (seconds) as a function of the number of spots per slice. We simulated two-dimensional spatial transcriptomics datasets with increasing numbers of spatial spots per slice, ranging from 1,500 to 19,000. Consistently with the nature of optimal transport computations, the trend in the plot suggests that the runtime grows approximately with the square of the number of spots, indicating a quadratic time complexity. Empirically, we observed that the runtime remains under one minute for datasets with fewer than 5,000 spots and increases to approximately 7.5 minutes for datasets with nearly 20,000 spots. All timing results were obtained using a single CPU core, without GPU acceleration. To further underscore real-world scalability, we applied TOAST to the integration of scRNA-seq and Visium HD slices[1], confirming that TOAST operates efficiently on high-resolution datasets containing hundreds of thousands of spatial spots.

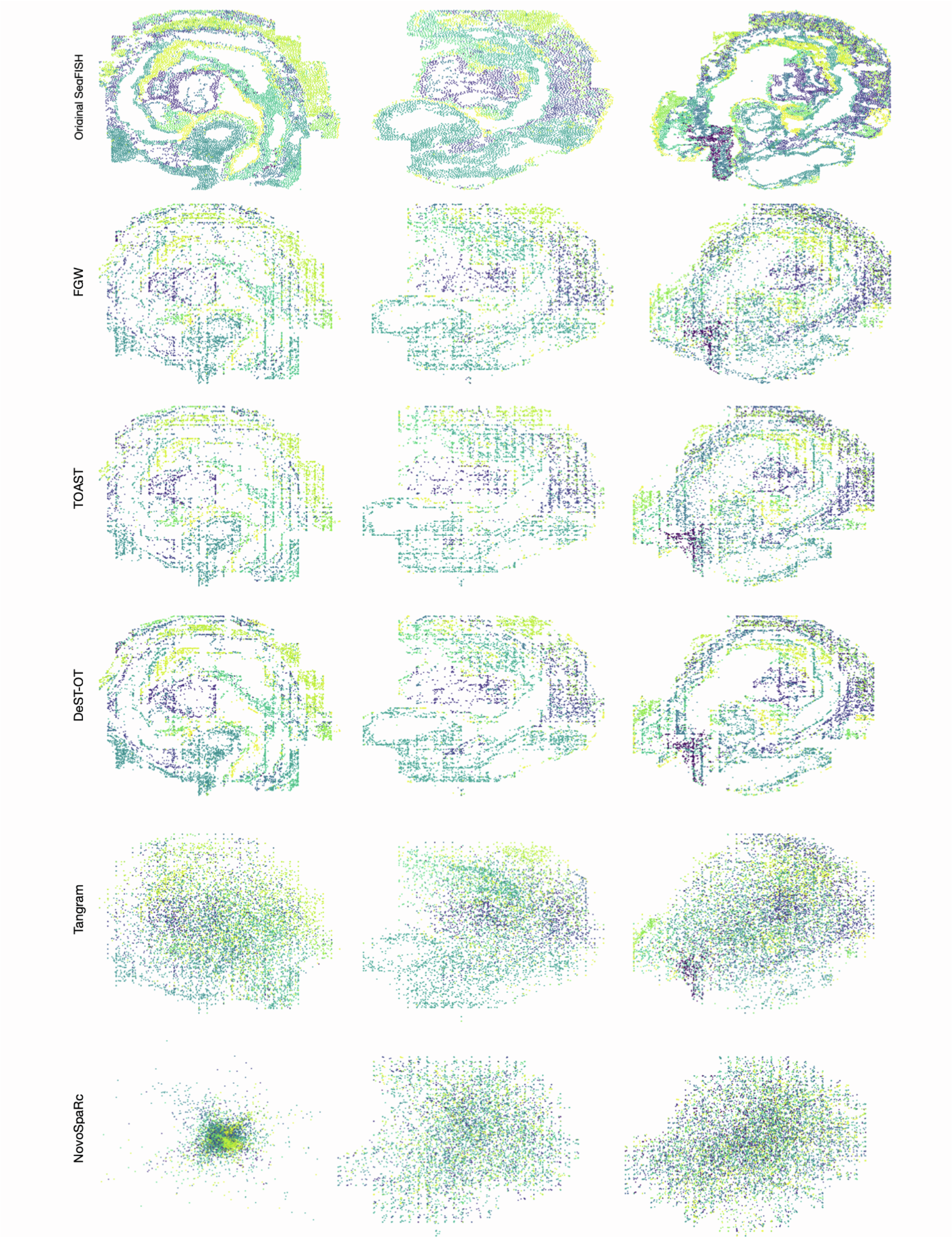

Figure S4: Spatial reconstruction on Mouse Atlas data, related to **Figure 6.** The reconstructed spatial distribution by FWG, TOAST, DeST-OT, Tangram and NovoSparc versus the ground truth spatial distribution. Colors indicate different cell types or regions.

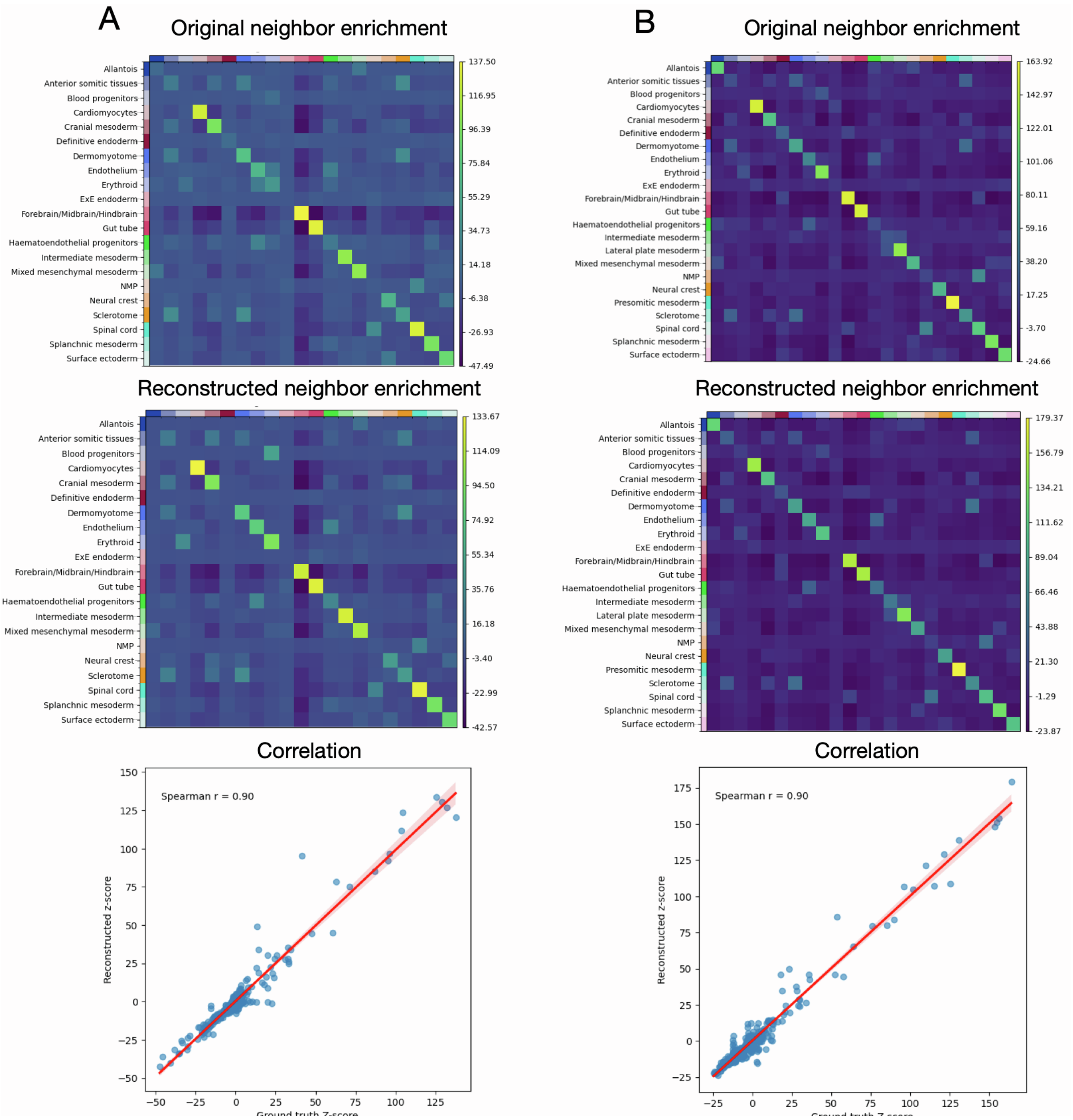

Figure S5: Neighborhood enrichment for individual samples from the Mouse Atlas dataset, related to **Figure 7**. **A.)** Heatmaps showing the neighbor enrichment scores for different cell types in the original slice **(top)**, the reconstructed slice **(middle)**, and correlation between reconstructed and ground truth z-scores **(bottom)** for sample E1z2 and **B.)** E1z3 from the Mouse Atlas.

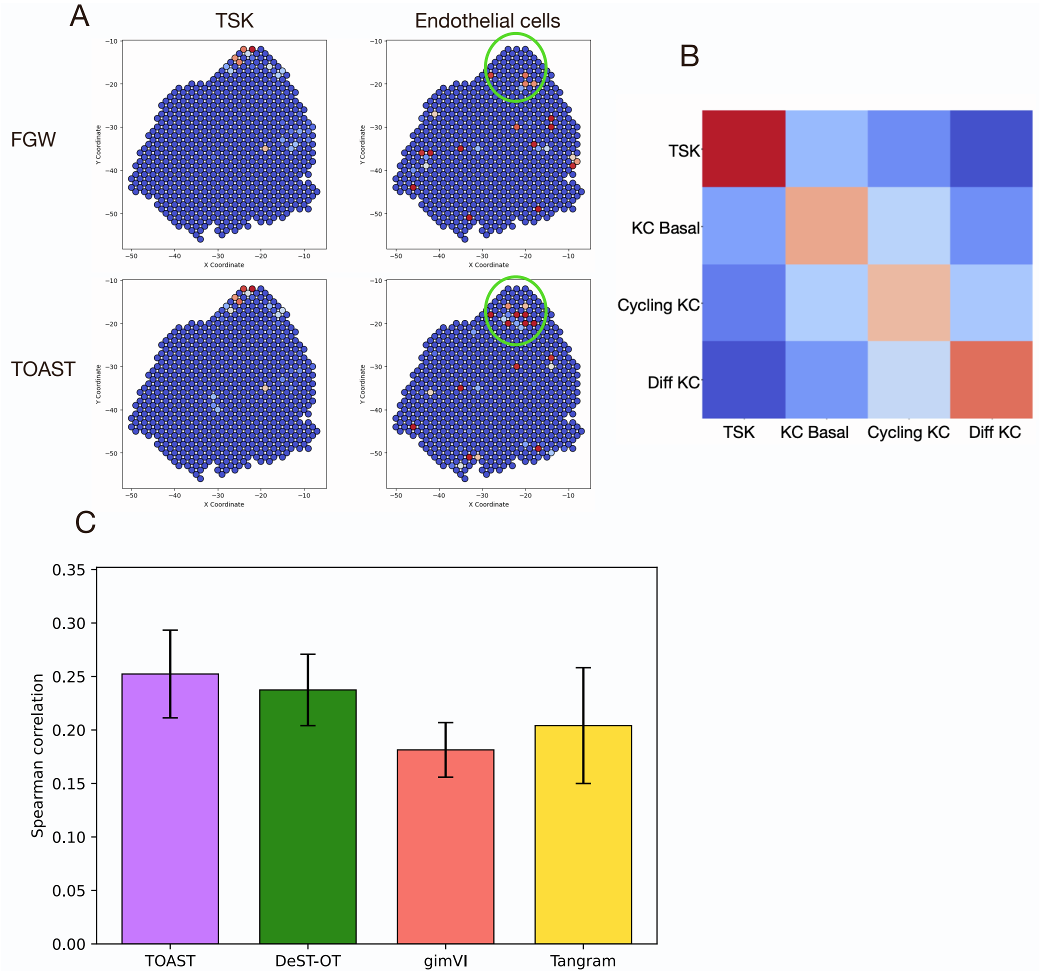

Figure S6: Human squamous cell carcinoma (hSCC) and gene expression imputation, related to **Figure 7**. **A.)** Cell type distribution reconstruction by FGW and TOAST for one donor from the human squamous cell carcinoma (hSCC) dataset[2]. While both FGW and TOAST identified tumor specific keratinocytes, FGW fails to identify the enrichment of endothelial cells closer to the tumor region described in the original publication. **B.)** Neighbor enrichment analysis on tumor keratinocyte subtypes: tumor specific keratinocytes (TSKs) tended to self-aggregate and spatially separate from other KCs. These findings, consistent with those of the original study, are crucial in revealing the spatial characteristics of the tumor microenvironment. **C.)** Comparison of Spearman correlation between predicted and ground-truth spatial gene expression values for held-out genes in the mouse somatosensory cortex dataset across five runs. TOAST achieves higher correlation than DeST-OT, gimVI and Tangram, indicating improved accuracy in unmeasured gene prediction.

References

1. Ciervo, E., Ceccarelli, F., Di Giacomo, A.M., Grisolia, P., Covre, A., Besharat, Z.M., De Falco, A., Caruso, F.P., Laezza, L., Ferraro, L., et al. (2025). Transposable Elements and Homotypic Niches Drive Immune Dynamics and Resistance in Melanoma Epigenetic-based immunotherapy. bioRxiv. https://doi.org/10.1101/2025.10.09.679175.

2. Ji, A.L., Rubin, A.J., Thrane, K., Jiang, S., Reynolds, D.L., Meyers, R.M., Guo, M.G., George, B.M., Mollbrink, A., Bergenstråhle, J., et al. (2020). Multimodal analysis of composition and spatial architecture in human squamous cell carcinoma. Cell *182*, 497–514.
